# RAD51 paralogs synergize with RAD51 to protect reversed forks from cellular nucleases

**DOI:** 10.1093/nar/gkad856

**Published:** 2023-10-16

**Authors:** Chia-Lun Guh, Kai-Hang Lei, Yi-An Chen, Yi-Zhen Jiang, Hao-Yen Chang, Hungjiun Liaw, Hung-Wen Li, Hsin-Yung Yen, Peter Chi

**Affiliations:** Institute of Biochemical Sciences, National Taiwan University, Taipei, Taiwan; Institute of Biochemical Sciences, National Taiwan University, Taipei, Taiwan; Institute of Biological Chemistry, Academia Sinica, Taipei, Taiwan; Institute of Biochemical Sciences, National Taiwan University, Taipei, Taiwan; Institute of Biochemical Sciences, National Taiwan University, Taipei, Taiwan; Department of Chemistry, National Taiwan University, Taipei, Taiwan; Department of Life Sciences, National Cheng Kung University, Tainan, Taiwan; Department of Chemistry, National Taiwan University, Taipei, Taiwan; Institute of Biochemical Sciences, National Taiwan University, Taipei, Taiwan; Institute of Biological Chemistry, Academia Sinica, Taipei, Taiwan; Institute of Biochemical Sciences, National Taiwan University, Taipei, Taiwan; Institute of Biological Chemistry, Academia Sinica, Taipei, Taiwan

## Abstract

Fork reversal is a conserved mechanism to prevent stalled replication forks from collapsing. Formation and protection of reversed forks are two crucial steps in ensuring fork integrity and stability. Five RAD51 paralogs, namely, RAD51B, RAD51C, RAD51D, XRCC2 and XRCC3, which share sequence and structural similarity to the recombinase RAD51, play poorly defined mechanistic roles in these processes. Here, using purified BCDX2 (RAD51BCD-XRCC2) and CX3 (RAD51C-XRCC3) complexes and *in vitro* reconstituted biochemical systems, we mechanistically dissect their functions in forming and protecting reversed forks. We show that both RAD51 paralog complexes lack fork reversal activities. Whereas CX3 exhibits modest fork protection activity, BCDX2 significantly synergizes with RAD51 to protect DNA against attack by the nucleases MRE11 and EXO1. DNA protection is contingent upon the ability of RAD51 to form a functional nucleoprotein filament on DNA. Collectively, our results provide evidence for a hitherto unknown function of RAD51 paralogs in synergizing with RAD51 nucleoprotein filament to prevent degradation of stressed replication forks.

## Graphical Abstract

**Graphical Abstract:**
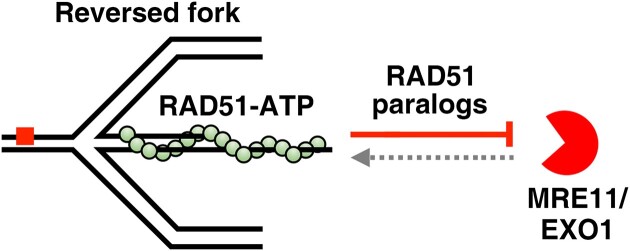

## Introduction

Faithful and complete DNA replication is crucial for maintaining genome integrity. During DNA replication, elongating forks may encounter various endogenous and exogenous insults, leading to fork stalling. Unresolved stalled forks may give rise to fork collapse and DNA double-strand breaks (DSBs) (1,2). Replication-induced DSBs can be repaired by break-induced replication (BIR), which is error-prone and can lead to genome instability (3). Cells possess other rescue pathways to resolve stalled replication forks, including PrimPol-mediated repriming and synthesis, translesion DNA synthesis (TLS) and fork reversal (4,5). The PrimPol polymerase initiates new DNA replication downstream of a DNA lesion, and TLS polymerases can replicate a damaged DNA template or fill the DNA gap left by repriming (4,5). However, both Primpol and TLS polymerases are error-prone since they lack proofreading activity (6,7). In contrast, the fork reversal pathway mediates a template-switching process that bypasses a DNA lesion in an error-free manner (4,8).

In fork reversal, a three-way stalled fork is remodeled into a four-way junction, which actively restrains DNA replication in unfavorable conditions, thus avoiding fork collapse (2,8,9). The reversed fork can then restart replication via the following mechanisms: (i) the reversed fork acts as a stabilized fork structure, allowing more time for the damaged DNA template to be repaired; (ii) a nearby origin firing and a replication fork merge with a reversed fork to rescue replication or (iii) the newly synthesized nascent DNA can provide an undamaged template for DNA synthesis to bypass the DNA lesion, termed the ‘template-switching mechanism’ (4,10,11).

Although fork regression is a valuable mechanism for bypassing DNA lesions, reversed forks are prone to attack by cellular nucleases, including MRE11, EXO1 and DNA2 (12–15). Proteins that function in homologous recombination (HR)—including BRCA1, BRCA2, RAD51, and CST—can protect the reversed forks from being extensively degraded by the aforementioned nucleases (16–20). Thus, replication fork regression must be coupled with stressed fork protection mechanisms to achieve the desired outcome of fork preservation.

RAD51, the recombinase that mediates DNA strand invasion during HR, is a prerequisite for regulating the dynamics of reversed forks (18,19,21). More specifically, the depletion of RAD51 causes a significant decrease in the number of reversed forks (18,21), indicating that RAD51 is indispensable for fork reversal. A recent report demonstrated that RAD51 promotes fork reversal by its strand-exchange activity to bypass CMG helicase (22). Moreover, *in vitro* reconstitution assays and DNA fiber analysis have revealed that RAD51 protects reversed forks (18,19,23). Notably, the separation-of-function RAD51 T131P mutant variant, which lacks the ability to form a stable nucleoprotein filament (24), can still generate reversed forks but lacks fork protection activity, indicating that a stable RAD51 nucleoprotein filament is likely indispensable for fork protection (18,19). Thus, existing evidence indicates that RAD51 functions in both the formation and protection of reversed forks.

RAD51 paralogs, encompassing a family of five proteins showing 20–30% sequence similarity with RAD51, also participate in fork preservation under replication stress (25). These five RAD51 paralogs—RAD51B, RAD51C, RAD51D, XRCC2 and XRCC3—form two distinct complexes, i.e. BCDX2 (RAD51B–RAD51C–RAD51D–XRCC2) and CX3 (RAD51C–XRCC3) (25–27). All RAD51 paralogs possess Walker A and B motifs, and the BCDX2 complex possesses single-stranded DNA (ssDNA)-stimulated ATPase activity (26,28). In addition, both BCDX2 and CX3 bind ssDNA and partial duplex DNA with either a 5′ or 3′ single-stranded overhang. Whereas CX3 can bind double-stranded DNA (dsDNA), BCDX2 lacks that ability (25–28).

Genetic knockout of each RAD51 paralog in mouse models results in embryonic lethality (29–33), and depletion of RAD51C, RAD51D, XRCC2 or XRCC3 from the human MCF10A cell line results in cell death (34). Notably, mono-allelic mutations in the RAD51 paralogs have been linked to various cancers and Fanconi anemia-like disorder (35–39). Previous research has primarily focused on the physiological role of RAD51 paralogs in regulating the RAD51-mediated HR repair pathway (34,40–43). Interestingly, accumulating evidence also indicates that RAD51 paralogs function in fork reversal to promote the restart of stalled replication forks (44–48). RAD51 paralogs are found to localize at challenged replication forks (44,47) and act in both fork reversal (46) and protection (44,45,47,48). However, the precise roles of RAD51 paralogs in regulating reversed fork dynamics remain controversial. Various arguments have been put forth in this regard, with Berti *et al.* suggesting that RAD51 paralogs enhance the formation, but not protection, of reversed forks (46), and Saxena *et al.* (2018) showing that depletion of RAD51 paralogs elicits severe fork degradation (45). Given the multiple functions of RAD51 paralogs in both reversed fork formation and downstream fork protection, dissecting their specific roles in replication stress by means of cellular studies can be challenging.

Using highly purified BCDX2 and CX3, here, we address the possible functional roles of these protein complexes in replication fork reversal and protection. We show that neither BCDX2 nor CX3 is able to promote fork reversal. Importantly, whereas CX3 exhibits modest fork protection activity, we uncover two attributes of BCDX2 that implicate it in fork protection, namely (i) that it modestly protects dsDNA from digestion by MRE11 and EXO1, and, importantly, (ii) that it significantly synergizes with RAD51 in DNA protection. Furthermore, we show that a functional RAD51 nucleoprotein filament is important for the synergistic protective effect. We analyze several disease-associated mutant variants of RAD51 and BCDX2 and find them to be defective in DNA protection. Therefore, our results provide evidence for a direct role of RAD51 paralogs in replication fork preservation. Our experimental system will be valuable for defining the impact of cancer-associated mutations in the RAD51 paralogs on replication fork protection and maintenance.

## Materials and methods

### Cloning, expression and purification of recombinant proteins

#### Human BCDX2 protein complex and its mutant variants

The RAD51B, RAD51C, RAD51D and XRCC2 cDNAs were cloned into a pcDNA3.4-tetrad vector with a Flag tag and a hexahistidine (His_6_) tag added to the amino terminus of RAD51C and XRCC2, respectively. In addition, a Tobacco etch virus (TEV) cutting site was added between the tags and the genes. To express the BCDX2 protein complex, the pcDNA3.4-tetrad-BCDX2 plasmid was transfected into Expi293F cells (Thermo Fisher) according to the instruction manual from the ExpiFectamine 293 kit (Thermo Fisher). The cells were harvested by centrifugation 48 h after transfection. The subsequent purification steps were carried out at 4°C. The harvested cells were suspended in buffer A (25 mM Tris–HCl, pH 7.5, 10% glycerol, 500 mM KCl, 0.05% Igepal-CA-630, 0.5 mM EDTA, 10 mM ATP, and 10 mM MgCl_2_) supplemented with 1 mM β-mercaptoethanol and protease inhibitors (2 mM PMSF, 2 mM Benzamidine and 1 μg/ml each of aprotinin, chymostatin, leupeptin, and pepstatin A). The cells were then lysed by sonication, followed by ultra-centrifugation at 100 000 × g for 1 h, to obtain the soluble extract containing target proteins. The supernatant was incubated with Ni-NTA agarose resin (QIAGEN) for 3 h. The resin was washed with buffer A supplemented with 10 mM imidazole and with buffer B (25 mM Tris–HCl, pH 7.5, 10% glycerol, 300 mM KCl, 0.05% Igepal-CA-630, 0.5 mM EDTA, 10 mM ATP and 10 mM MgCl_2_) supplemented with 20 mM imidazole. The BCDX2 protein complex was eluted with buffer B supplemented with 200 mM imidazole. The eluate was then loaded into anti-Flag M2 affinity agarose (Sigma Aldrich), and the protein-bound resin was washed with buffer B. The BCDX2 protein complex was eluted with buffer B supplemented with 3× Flag peptide (100 μg/ml). The eluate was then loaded on a Superdex 200 Increase 10/300 GL column (GE Healthcare) equilibrated with buffer C (25 mM Tris–HCl, pH 7.5, 10% glycerol, 0.05% Igepal-CA-630, 1 mM β-mercaptoethanol, 0.5 mM EDTA) supplemented with 300 mM KCl. The soluble fractions (151 kDa) were pooled, concentrated, aliquoted and stored at –80°C.

The BCDX2 expression plasmids containing the RAD51C Q133K or C135Y mutant variant were generated by site-directed mutagenesis. The expression and purification procedure of those mutant variants were as described above for the BCDX2 wild-type complex. It is worth noting that RAD51C C135Y mutation leads to unstable complex formation. Thus, purification was performed with buffers containing lower salt (75 mM KCl) to avoid disassembly of the protein complex.

#### Human CX3, BC and DX2 protein complex

The cDNA of RAD51C with a hexahistidine (His_6_) tag followed by a Flag tag at the amino terminus and XRCC3 cDNA were cloned into a pcDNA3.4-dual vector to obtain the CX3 expression plasmid. RAD51B and the hexahistidine (His_6_)- and Flag-tagged RAD51C sequence were cloned into a pcDNA3.4-dual vector to generate the BC expression plasmid. The cDNA of RAD51D and the hexahistidine (His_6_)- and Flag-tagged XRCC2 sequence were cloned into a pcDNA3.4-dual vector to construct the DX2 expression plasmid. To express the CX3, BC and DX2 complexes, Expi293F cells were transiently transfected with the individual plasmid and harvested by centrifugation 48 h post-transfection. The cells were suspended, lysed, and centrifuged to obtain the cell extract as described above. The cell extract was then incubated with Ni-NTA agarose resin for 3 h. The resin was washed with buffer A supplemented with 10 mM imidazole, and with buffer B supplemented with 20 mM imidazole. The target protein complexes were eluted with buffer B supplemented with 200 mM imidazole. The eluate was then loaded into anti-Flag M2 affinity agarose, and the protein-bound resin was washed with buffer B. The desired protein complex was eluted with buffer B supplemented with 3x Flag peptide (100 μg/ml). The eluate was then loaded on a Superdex 200 Increase 10/300 GL column equilibrated with buffer C supplemented with 300 mM KCl. The soluble fractions were pooled, concentrated, aliquoted, and stored at –80°C.

#### Human SMARCAL1 recombinant protein

The cDNA of SMARCAL1 was cloned into a pcDNA3.4 vector with a hexahistidine (His_6_) tag and a Flag tag, followed by a tobacco etch virus (TEV) cutting site added to the amino terminus of the protein. To express the SMARCAL1 protein, Expi293F cells were transiently transfected with the expression plasmid and harvested by centrifugation 48 h post-transfection. The SMARCAL1-expressing cells were then suspended, lysed, and centrifuged as described above. The cell lysate containing the His_6_-Flag-SMARCAL1 recombinant protein was purified by Ni-NTA agarose resin, anti-Flag M2 agarose affinity pulldown, and a Superdex 200 Increase 10/300 GL column as described above, except that the buffers contained 0.01% Igepal-CA-630. The soluble fractions were pooled, concentrated, aliquoted and stored at –80°C.

#### Human MRE11 recombinant protein

Human MRE11 was expressed and purified as described previously (20). In brief, Flag-MRE11-His_6_ recombinant protein was purified by Ni-NTA agarose resin and anti-Flag M2 affinity agarose affinity pulldown using a 1 ml Mono Q column (GE Healthcare). The fractions containing pure MRE11 were pooled, concentrated, aliquoted, and stored at –80°C.

#### Human EXO1 recombinant protein

The cDNA of EXO1b (purchased from Addgene, catalog #111621) was cloned into a pcDNA3.4 vector with a Flag tag added to the carboxyl-terminus of EXO1, generating the pcDNA3.4-EXO1-Flag expression plasmid. The EXO1 expression plasmid was transfected into Expi293F cells, and the EXO1-expressing cells were harvested. The harvested cells were suspended, lysed, and centrifuged to obtain the cell extract as described above. The cell lysate was incubated with anti-Flag M2 affinity agarose for 1 h, and the protein-bound resin was then washed with buffer B. The EXO1 protein was eluted with buffer B supplemented with 3x Flag peptide (100 μg/ml). The eluate was then fractionated with a 1 ml Heparin column (GE Healthcare), using a 30 ml gradient of 100–640 mM KCl in buffer C. The EXO1-containing fractions were collected and loaded on a 1 ml HiTrap SP HP column (GE Healthcare) and fractionated with a 5 ml gradient of 100–1000 mM KCl in buffer C. The EXO1 fractions were then loaded on a Superdex 200 Increase 10/300 GL column equilibrated with buffer C supplemented with 300 mM KCl. The soluble fractions were pooled, concentrated, aliquoted, and stored at –80°C.

#### Human RAD51 and its mutant variants

Human RAD51 protein was purified as described previously (49). The RAD51 K133A, K133R, II3A and Y232W mutant variants were expressed and purified as that for the RAD51 wild-type protein. For the RAD51 T131P mutant variant, the cDNA of RAD51 T131P generated by site-directed mutagenesis was cloned into a pRSFDuet-His-SUMO plasmid. The His-SUMO-RAD51 T131P protein was expressed in the *Escherichia coli* RecA-deficient strain BLR as described for the wild-type protein. The cell pellet was suspended in buffer D (20 mM K_2_HPO_4_, pH 7.5, 10% glycerol, 0.01% Igepal-CA-630, 1 mM β-mercaptoethanol) supplemented with 300 mM KCl and protease inhibitors for sonication and ultracentrifugation. The cell extract was incubated with Talon resin (Clontech) for 3 h. The resin was washed first with buffer D supplemented with 300 mM KCl and 10 mM imidazole, and next with buffer D supplemented with 150 mM KCl and 10 mM imidazole. To obtain tag-free RAD51 T131P, strep-Ulp1 protease was mixed with the resin for 2 h to cleave the His-SUMO tag on RAD51 T131P. The supernatant containing non-tagged RAD51 T131P was loaded on a 8 ml Source Q column and fractionated using a 135 ml gradient of 193–715 mM KCl in buffer D supplemented with 0.5 mM EDTA. The RAD51 T131P-containing fractions were then loaded on a 1 ml Heparin column (GE Healthcare) and fractionated with a 15 ml gradient of 145–810 mM KCl in buffer D supplemented with 0.5 mM EDTA. At last, the RAD51 T131P-containing fractions were further fractionated using a 1 ml Mono Q column with a 15 ml gradient of 288–573 mM KCl in buffer D supplemented with 0.5 mM EDTA. Fractions containing RAD51 T131P were then pooled, filter-dialyzed into buffer C supplemented with 300 mM KCl, concentrated, aliquoted and stored at –80°C.

#### Other recombinant proteins

For *Saccharomyces cerevisiae* Rad51 protein (yRad51), the purification procedure was modified from a protocol described previously (50). In brief, the yRad51 protein was isolated sequentially by ammonium sulfate precipitation, followed by Sepharose Q column, macro hydroxyapatite, and Source Q column purification steps.

*Escherichia coli* RecA protein and ExoIII protein were purchased from New England Biolabs.

### Mass spectrometry analysis

To prepare MS samples for protein identification, the purified BCDX2 and CX3 complexes were resolved in a 12% SDS-PAGE gel, respectively, and visualized by Coomassie Blue staining. Individual bands were excised from stained gels, followed by reduction, alkylation, trypsin digestion, and extraction. An Orbitrap Elite^TM^ Hybrid Ion Trap-Orbitrap Mass Spectrometry was used for ESI-MS/MS spectrometry. The MS/MS signal was then analyzed with Mascot software.

### Native-mass spectrometry of the BCDX2 complex

Protein samples were buffer exchanged into 500 mM ammonium acetate buffer pH 7.5, loaded into gold-coated emitters fabricated in-house, and sprayed into a modified Q-Exactive mass spectrometer (Thermo Fisher Scientific). Typical MS parameters included spray voltage, 1.1 kV; capillary temperature, 200°C; S-lens RF level, 200; resolution, 12 500. To avoid collisional activation of the ions before transmission into the higher-energy collisional dissociation (HCD) cell, a gentle voltage gradient (injection flatapole, inter flatapole, bent flatapole, and transfer multipole: 5.0, 4.0, 2.0, 1.0 V, respectively), HCD energy of 5V, and a trapping voltage of 0V were applied. To dissociate protein complexes, in-source trapping was applied with desolvation voltages ranging between −50 and −250 V, and trapping gas pressure was set to a value of 3.0. RAW files were analyzed and processed manually using Thermo Xcalibur Qual Browser (version 4.4.16.14).

### DNA substrates

The DNA oligonucleotides used in this study are listed in Supplementary Table S3. The fluorescently-labeled DNA oligonucleotides were purchased from Genomics. The non-modified DNA oligonucleotides and the phosphorothioate bond-modified DNA oligonucleotides were purchased from Integrated DNA Technologies. To prepare the leading-strand gap substrate for fork reversal assay, oligos 1 and 2 were mixed in a ratio of 1:1.2 in annealing buffer (50 mM Tris pH 7.5, 10 mM MgCl_2_, 100 mM NaCl and 1 mM DTT). In parallel, oligos 3 and 4 were similarly mixed. The mixture was heated at 80°C for 3 min, then transferred to 65°C for 30 min, and cooled down slowly to room temperature in the water bath. The two respective mixtures (oligos 1 + 2 and 3 + 4) were subsequently mixed with an equal molar ratio and incubated at 37°C for 30 min. The annealed replication fork substrate was purified from an 8% TBE–PAGE gel by electro-elution, and the substrate was filter-dialyzed into TE buffer (10 mM Tris–HCl pH 8.0, and 0.5 mM EDTA) at 4°C using an Amicon ultra-4 concentrator (Millipore, NMWL 10 kDa).

The preparation of the lagging-strand gap substrate was similar to that of the leading-strand gap substrate. Oligos 1 and 5 were mixed in a ratio of 1:1.2, and the complementary half consisting of oligos 4 and 6 were mixed separately. As described above, the two respective substrates were further annealed with an equal molar ratio, gel-purified, concentrated, and filter-dialyzed into TE buffer to generate the lagging-strand gap substrate.

For nuclease degradation assays, except for the Cy3-labeled oligonucleotide, other DNA oligonucleotides were modified with phosphorothioate bonds on both ends to prevent non-specific nuclease digestion. Fluorescently-labeled oligo 7 was annealed with the phosphorothioate bond-modified oligo 8 to generate a 5′ overhang DNA substrate. Fluorescently-labeled oligo 9 was annealed with the phosphorothioate bond-modified oligo 8 to generate a 3′ overhang DNA substrate. The fluorescently-labeled reversed fork-like DNA substrate was prepared by mixing oligos 10, 11, 12 and 13 in the annealing buffer, further heated at 80°C for 3 min, then transferred to 65°C for 30 min, and cooled down slowly to room temperature in the water bath. All the annealed substrates were further gel-purified, concentrated and filter-dialyzed into TE buffer, as described above.

The double-stranded DNA substrate used in the DNA mobility shift assay was prepared by mixing oligos 13 and 14 in the annealing buffer, before transferring the mixture to 65°C for 30 min and cooling it down slowly to room temperature in a water bath. The annealed substrates were further gel-purified, concentrated and filter-dialyzed into TE buffer, as described above.

### Fork reversal assay

Fluorescently-labeled fork substrate (8 nM molecules) was incubated with indicated proteins in 10 μl buffer E (35 mM Tris–HCl, pH 7.5, 1 mM DTT, 0.1 μg/μl BSA, 2.5 mM MgCl_2_) supplemented with 100 mM KCl and 2 mM ATP at 37°C for 30 min. The reaction was then mixed with 2.5 μl termination buffer (50 mM EDTA, 0.1% SDS, and 3.2 mg/ml proteinase K) and incubated at 37°C for 15 min to stop the reaction. The reaction mixtures were resolved in a 6% TBE-PAGE gel with 1 × TBE buffer at 110 V for 70 min. Gels were analyzed using an Amersham™ Typhoon™ Biomolecular Imager with a Cy3 570BP20 560–580 nm filter. ImageJ software was then used to quantify the signal intensity of DNA species.

### Electrophoretic mobility shift assay

Fluorescently-labeled 5′ overhang, 3′ overhang, or double-stranded DNA substrate (58 nM molecules) was incubated with the indicated amount of BCDX2 or CX3 in 10 μl buffer E supplemented with 50 mM KCl and 1 mM ATP at 37°C for 10 min. The reaction mixtures were resolved in a 0.8% TAE-agarose gel with 0.5 × TAE buffer at 80 V for 70 min. Gels were analyzed using an Amersham™ Typhoon™ Biomolecular Imager with a Cy3 570BP20 560–580 nm filter.

Fluorescently-labeled fork substrate (8 nM molecules) was incubated with RAD51 in 10 μl buffer E supplemented with 100 mM KCl and 2 mM ATP at 37°C for 10 min. The reaction mixtures were resolved in a 6% TBE–PAGE gel with 1 × TBE buffer at 110 V for 70 min. Gels were analyzed using an Amersham™ Typhoon™ Biomolecular Imager with a Cy3 570BP20 560–580 nm filter.

### ATPase assay

BCDX2 or CX3 (1.58 μM) was incubated with 1 μCi [γ-^32^P]ATP in the presence or absence of single-stranded DNA (15.8 μM nucleotides, phiX174 virion) in 10 μl buffer E supplemented with 50 mM KCl and 50 μM ATP for the indicated times. Aliquots of 2 μl were withdrawn at the indicated times and mixed with an equal volume of 500 mM EDTA to stop the reaction. The level of ATP hydrolysis was determined by thin-layer chromatography on polyethyleneimine cellulose sheets (MACHEREY-NAGEL, 801063) developed in 0.5 M LiCl and 1M formic acid, with phosphorimaging analysis conducted in a Personal FX phosphorimager using the Quantity One software (Bio-Rad).

### MRE11 protection assay

Fluorescently-labeled 5′ overhang DNA substrate (58 nM molecules) or fluorescently-labeled reversed fork-like DNA substrate (29 nM molecules) was incubated with the indicated amount of specified proteins and purified human MRE11 in 10 μl buffer E supplemented with 50 mM KCl, 1 mM ATP and 1 mM MnCl_2_ at 37°C for the indicated time. The reaction was then mixed with 2.5 μl termination buffer and incubated at 37°C for 15 min to stop the reaction. An equal volume of 2× denature dye (95% formamide, 0.1% Orange G, 10 mM Tris–HCl, pH 7.5, 1 mM EDTA, and 12% Ficoll PM400) was added into the reaction mixture and incubated at 95°C for 10 min to denature the DNA substrates. The samples were analyzed on 27% denature TBE-Urea-PAGE (7 M Urea) with 1 × TBE buffer at 300 V for 40 min at 55°C. Gels were analyzed using an Amersham™ Typhoon™ Biomolecular Imager with a Cy3 570BP20 560–580 nm filter. ImageJ software was then used to quantify the intensity of DNA species. The intensity of fully protected DNA species was normalized with the intensity of DNA substrate in control experiments to obtain the percentage of protection.

### EXO1 protection assay

Fluorescently-labeled 3′ overhang DNA substrate (58 nM molecules) was incubated with the indicated amount of specified proteins, including EXO1, in 10 μl buffer E supplemented with 50 mM KCl and 1 mM ATP at 37°C for 40 min. Subsequent reaction termination and resolution of the various DNA species through electrophoresis were as described above.

### ExoIII protection assay

Fluorescently-labeled 5′ overhang DNA substrate (58 nM molecules) was incubated with the indicated amount of the specified proteins and *E. coli* ExoIII in 10 μl buffer E supplemented with 50 mM KCl and 1 mM ATP at 37°C for 40 min. Subsequent reaction termination and resolution of the various DNA species by means of electrophoresis were as described above.

### Statistics

All statistical tests were performed using GraphPad Prism 7 (GraphPad software) as indicated to establish statistical significance. The Shapiro–Wilk test was used to examine the normality of the data. The sample variance was confirmed to be similar between groups by the Brown–Forsythe test for multiple groups. One-way ANOVA with Tukey's post hoc test was used for comparisons of multiple groups to determine statistical significance. A *P* value <0.05 was considered significant.

## Results

### Expression and purification of BCDX2 and CX3 complexes from human cells

**Figure 1.**
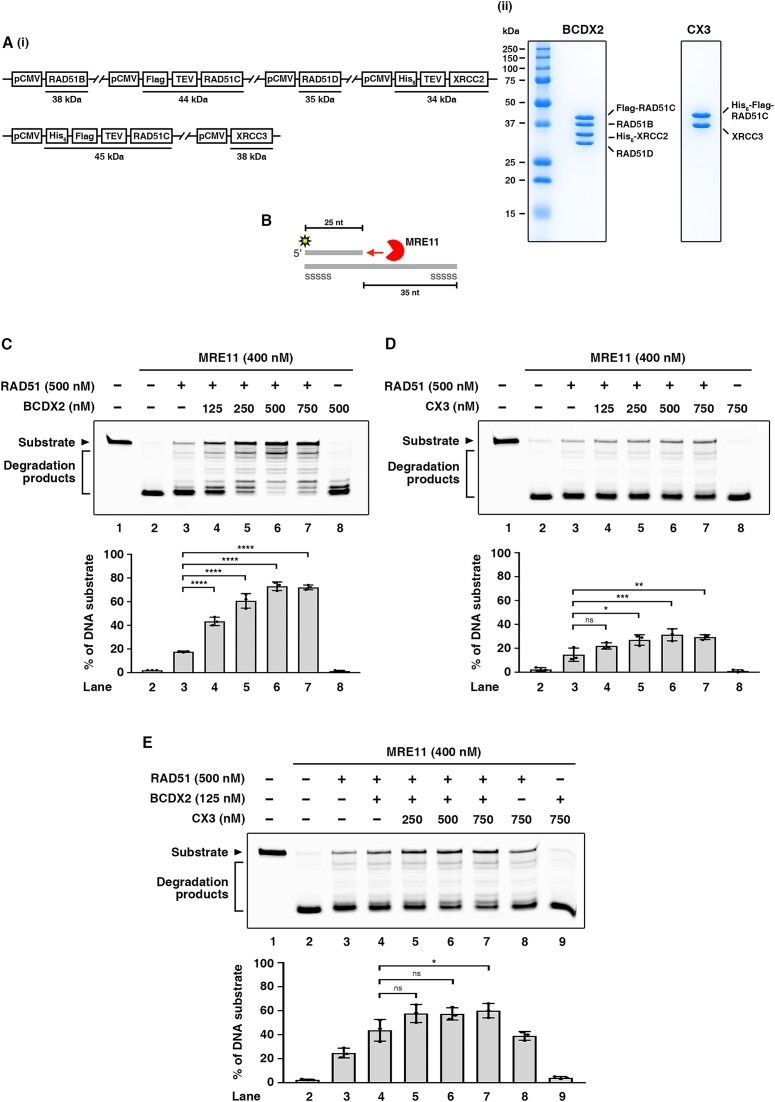
Purified BCDX2 synergizes with RAD51 to protect DNA against MRE11 nucleolytic degradation. (**A**) Expression constructs and purified proteins of the BCDX2 and CX3 complexes. (i) Schematics of the BCDX2 and CX3 expression constructs. (ii) The purified BCDX2 and CX3 complexes (2.5 and 1.25 μg, respectively) were analyzed in a 12% SDS-denaturing polyacrylamide gel with Coomassie Blue staining. (**B**) Illustration of a MRE11 protection assay on a 5′ overhang DNA substrate designed to mimic the regression arm of a reversed fork. The 5′ overhang DNA substrate is composed of a 5′ Cy3-labeled 25 nucleotide (nt) oligonucleotide and a 60 nt oligonucleotide with a phosphorothioate bond modification on both ends to avoid nonspecific nucleolytic degradation. (**C** and **D**) MRE11 protection assay. BCDX2 (**C**) or CX3 (**D**) at the indicated concentration, RAD51, and MRE11 were incubated with 58 nM Cy3-labeled 5′ overhang DNA substrate at 37°C for 40 min. Then the reactions were terminated and resolved in a 27% denatured polyacrylamide gel. (**E**) BCDX2, CX3 at the indicated concentration, RAD51, and MRE11 were incubated with 58 nM Cy3-labeled 5′ overhang DNA substrate at 37°C for 40 min. The reactions were then stopped and resolved as described above. For (C–E), the top panel is a representative gel image, and the bottom panel shows quantitative data (mean ± SD) calculated from at least three independent repeats. Statistical significance was determined using one-way ANOVA with Tukey's post hoc test; ns not significant, *P < 0.05, **P < 0.01, ***P < 0.001, ****P < 0.0001.

The two human RAD51 paralog complexes, BCDX2 and CX3, were expressed and purified from human cells to avoid protein misfolding and severe protein aggregation. In brief, the cDNA for each of the salient RAD51 paralogs was cloned into the same vector (Figure 1A, panel i) for expression in human Expi293F cells, with ATP-MgCl_2_ being included throughout the purification procedure to enhance the solubility of the protein complexes (51). Both BCDX2 and CX3 were purified to near homogeneity through a two-affinity and size exclusion purification procedure (Figure 1A, panel ii, see Materials and Methods for details). The identity of both BCDX2 and CX3 complexes was confirmed by liquid chromatography–tandem mass spectrometry (LC–MS/MS) (Supplementary Table S1). Both purified protein complexes were confirmed to be active in DNA binding, with BCDX2 also displaying ssDNA-stimulated ATP hydrolysis activity (Supplementary Figure S1), consistent with previous studies (26–28). Notably, we report here that CX3 also exhibits DNA-stimulated ATPase activity (Supplementary Figure S1C).

### BCDX2 and CX3 lack intrinsic fork reversal activity

Depletion of RAD51C from U2OS cells was shown previously to elicit a significant decrease in the number of reversed forks even upon MRE11 inhibition (46), indicating a potential role for RAD51 paralogs in facilitating the formation of reversed forks. We examined if BCDX2 and CX3 possess fork reversal activity on DNA substrates mimicking stalled replication forks with a ssDNA gap on either the leading or lagging strand (Supplementary Figure S2A). While the well-known fork remodeler SMARCAL1 efficiently converted either the leading- or lagging-strand gap fork into duplex products (Supplementary Figures S2B-S2G), no fork reversal activity could be detected for BCDX2 or CX3 (Supplementary Figure S2B and C).

RAD51 is required for the fork reversal process (21), and RAD51 paralogs are known as regulators of RAD51 function (34,40,41,52,53). Moreover, the amount of RAD51 required for promoting fork reversal is lower than that in protecting reversed forks (54). Therefore, we examined if RAD51 functions with BCDX2 and CX3 to catalyze fork reversal using various RAD51 concentrations. We first conducted an electrophoretic mobility shift assay that tested both low (40 nM) and relatively high (400 nM) concentrations of RAD51 and confirmed that a slight DNA shift occurred at 40 nM RAD51 and a complete DNA shift at 400 nM RAD51 (Supplementary Figure S3). With the two chosen concentrations of RAD51, we further include BCDX2 and CX3 in the reaction to see if fork reversal occurs. Importantly, neither BCDX2- nor CX3-mediated fork reversal was observed at either concentration of RAD51 (Supplementary Figures S2D–G).

### RAD51 paralogs synergize with RAD51 to protect DNA from MRE11 nucleolytic degradation

Depletion of RAD51 paralogs was found previously to result in severe fork degradation (45), which could be prevented by MRE11 inhibition (44). Therefore, we wondered if purified BCDX2 and CX3 could prevent MRE11-mediated DNA degradation *in vitro* of a substrate harboring a 5′ single-stranded overhang to mimic the regression arm of a reversed fork (Figure 1B). We found that BCDX2 possesses modest protective activity against MRE11 nucleolytic degradation, but CX3 is devoid of any such activity (Supplementary Figures S4A and B). Given that RAD51 is also involved in protecting reversed forks (18,19,23), we then explored if RAD51 paralogs act together with RAD51 to protect the nascent DNA. Though RAD51 alone exerted a protective effect on DNA, the addition of BCDX2 led to significant synergy in DNA protection (Figure 1C). Indeed, we detected a 4-fold increase in the amount of fully protected DNA at the highest concentration of BCDX2 we tested (Figure 1C). In contrast, only a modest increase in DNA protection occurred upon addition of CX3 with RAD51 or for CX3 plus RAD51–BCDX2 (Figures 1D and E). These results indicate that both of the RAD51 paralog complexes participate in RAD51-mediated fork protection. However, BCDX2 exerts a more prominent contribution compared to CX3 in protective synergy with RAD51. Accordingly, we focused our remaining mechanistic assays on BCDX2.

Next, we verified if this BCDX2–RAD51-elicited protective effect could be observed for a DNA substrate mimicking the structure of a four-way reversed fork (Supplementary Figure S5A, panel i). The reversed fork-like DNA substrate was validated by means of restriction enzyme analysis (Supplementary Figure S5A, panel ii). In agreement with previous reports (19,55), the reversed fork-like DNA substrate was degraded by MRE11 in the 3′-to-5′ direction (Supplementary Figure S5A, panel iii). Then, we tested if BCDX2, alone or in combination with RAD51, could protect that DNA substrate from MRE11 degradation. Consistent with the results we obtained for the 5′ single-stranded overhang substrate (Supplementary Figure S4A), BCDX2 alone exerted a slightly protective effect on the reversed fork substrate (Supplementary Figure S5B) but, notably, BCDX2 in conjunction with RAD51 significantly enhanced DNA protection (Supplementary Figure S5C).

### Neither the BC nor DX2 sub-complexes exhibit a synergistic effect with RAD51

To address how the BCDX2 complex synergistically protects reversed forks with RAD51, we dissected the stoichiometry of the BCDX2 complex. Consistent with previous observations (26,51), our size exclusion analysis revealed an elution profile for BCDX2 close to that of the 158 kDa size standard, implying a monomeric nature for the complex (Supplementary Figure S6A). However, a recent mass photometric analysis observed BCDX2 mostly a dimer of heterotetramer (56). To precisely clarify the stoichiometric ratio of BCDX2, we subjected the purified BCDX2 complex to native mass spectrometry analysis to accurately calculate the mass of the full complex under high resolution. Signals close to the theoretical mass of a monomeric complex were observed at low activation energy (Supplementary Figure S6B). By elevating the in-source activation energy, signals assigned to RAD51B, RAD51C, RAD51D and XRCC2 monomers were observed individually, implying complex disassembly (Supplementary Figure S6C). With mass errors of <1.5% (Supplementary Table S2), these results confirm the 1:1:1:1 stoichiometric ratio of the BCDX2 complex. Intriguingly, two mass species of 82370.5 ± 1.2 Da and 68719.0 ± 0.1 Da, corresponding to the RAD51B–RAD51C and RAD51D–XRCC2 sub-complexes, respectively, were observed after collision-induced dissociation (Supplementary Figure S6C and Table S2). This dissociation pattern is consistent with the observed weaker interaction between RAD51C and RAD51D in yeast-two-hybrid analysis (57) and the identification of BC and DX2 sub-complexes (52,58–60), supporting the presence of BC and DX2 sub-complexes within the full complex.

**Figure 2.**
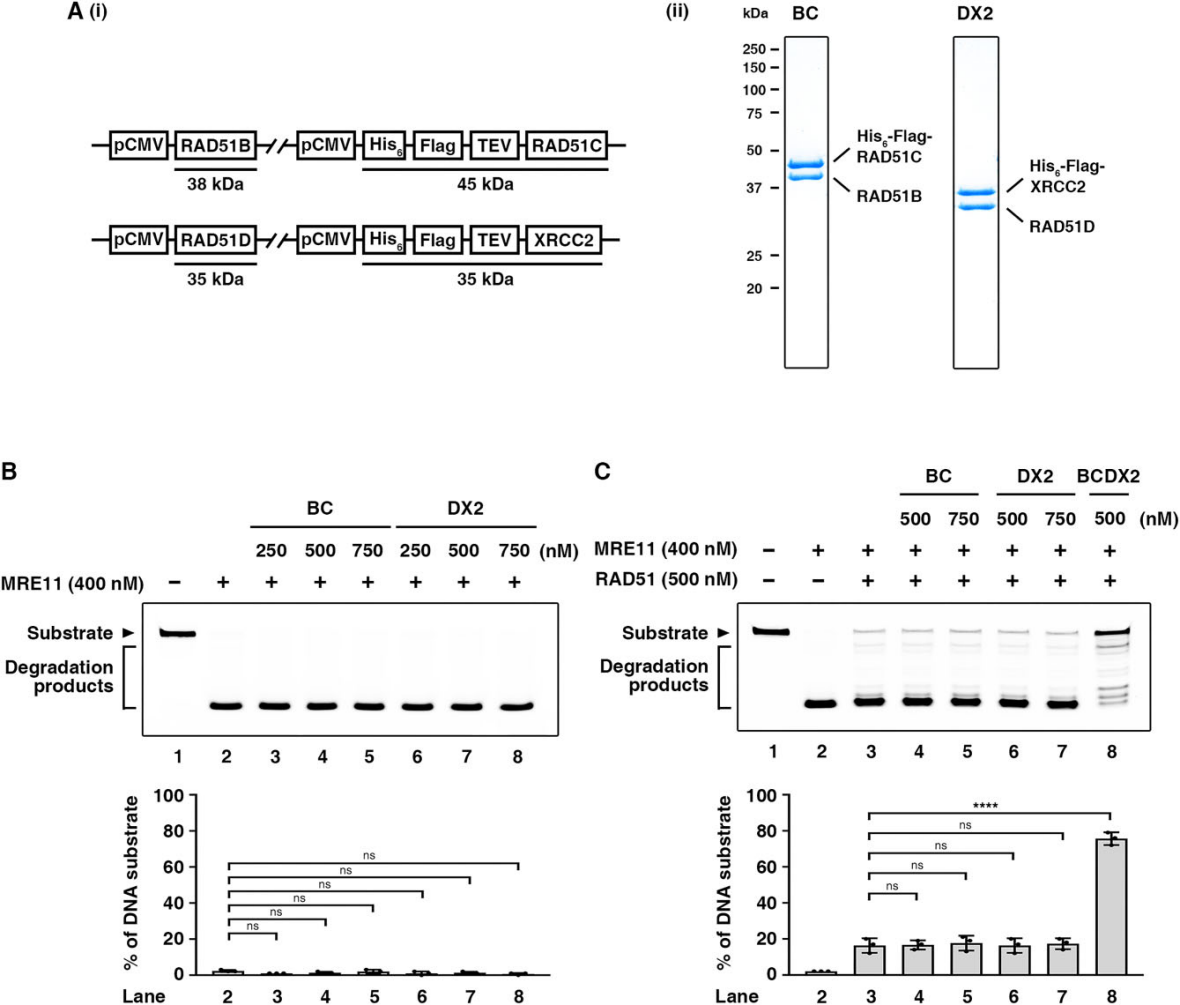
Neither the BC nor DX2 sub-complexes exhibit a synergistic effect with RAD51. (**A**) (i) Schematics of the BC and DX2 expression constructs. (ii) The purified BC and DX2 sub-complexes (1 μg of each complex) were analyzed in a 12% SDS-denaturing polyacrylamide gel with Coomassie Blue staining. (**B**) MRE11 protection assay. BC or DX2 sub-complexes at the indicated amounts and MRE11 were incubated with 58 nM Cy3-labeled 5′ overhang DNA substrate at 37°C for 40 min. (**C**) BC or DX2 sub-complexes at the indicated amounts, BCDX2, RAD51, and MRE11 were incubated with 58 nM Cy3-labeled 5′ overhang DNA substrate at 37°C for 40 min. For (B and C), the top panel is a representative gel image, and the bottom panel shows quantitative data (mean ± SD) calculated from at least three independent repeats. Statistical significance was determined using one-way ANOVA with Tukey's post hoc test; ns not significant, ****P < 0.0001.

Based on the data from our native mass spectrometry analysis, we expressed the BC and DX2 sub-complexes in human cells (Figure 2A, panel i) and purified both to a high degree of homogeneity (Figure 2A, panel ii). The biochemical activities of our purified BC and DX2 sub-complexes align with previously reported findings, demonstrating comparable patterns in DNA binding and ATP hydrolysis (61). To determine if BC and DX2 function in DNA protection, we examined the sub-complexes in a protection assay. Importantly, we observed that neither the BC nor DX2 sub-complex exhibited DNA protective activity against MRE11, regardless of the presence or absence of RAD51 (Figures 2B and C).

### Role of ATP in DNA protection by BCDX2-RAD51

**Figure 3.**
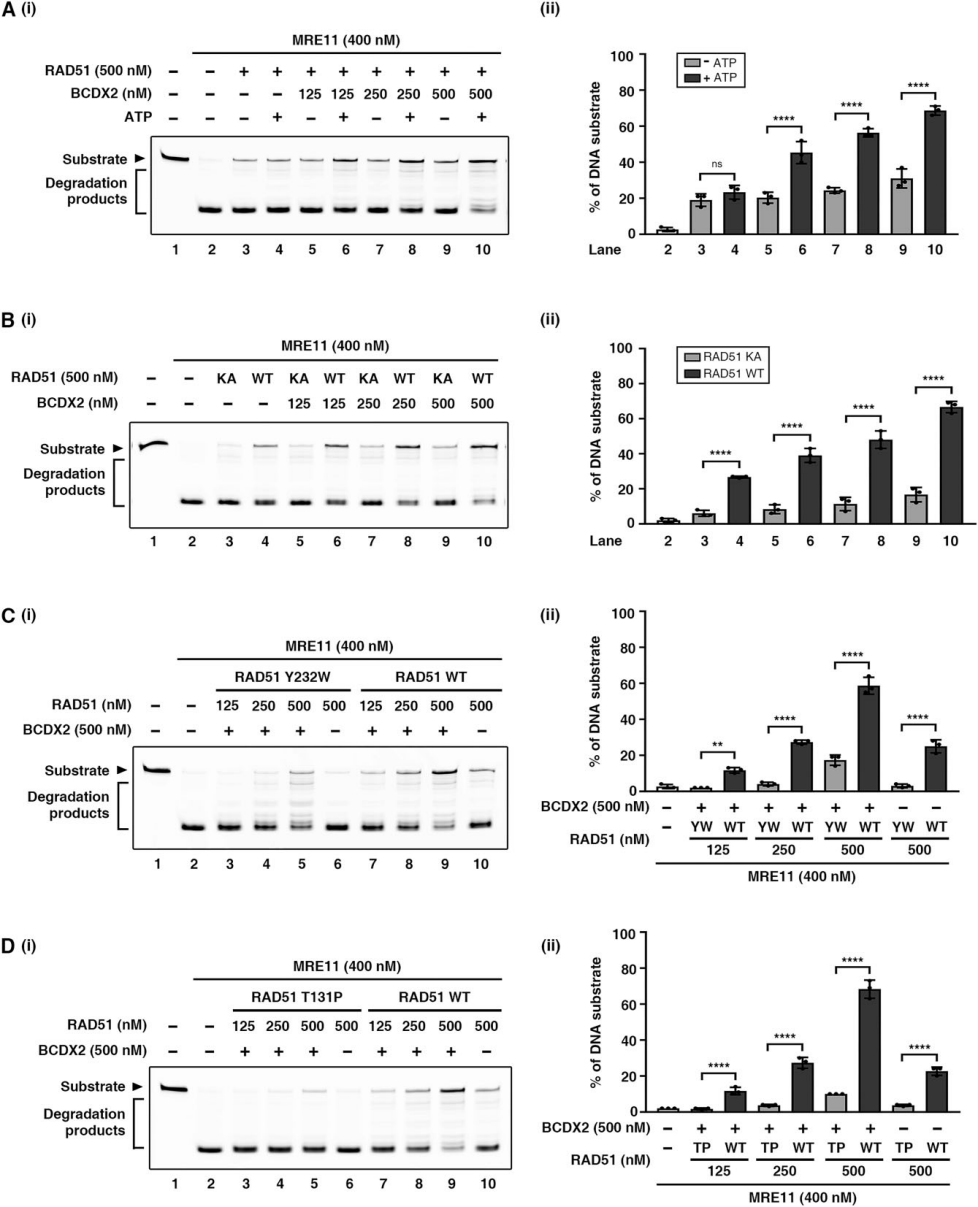
A functional RAD51 nucleoprotein filament is important for BCDX2-mediated synergistic DNA protection. (**A**) MRE11 protection assay in the presence or absence of 1 mM ATP. BCDX2 at the indicated concentration, RAD51, and MRE11 were incubated with 58 nM Cy3-labeled 5′ overhang DNA substrate at 37°C for 40 min. (**B**) MRE11 protection assay in the presence of the ATP-binding defective RAD51 K133A (KA) mutant. BCDX2 at the indicated concentration, RAD51 wild-type (WT) or KA, and MRE11 were incubated with 58 nM Cy3-labeled 5′ overhang DNA substrate at 37°C for 40 min. Note that 0.1 mM ATP was used in the reaction to ensure the RAD51 KA mutant variant lacked ATP-binding capability. (**C** and **D**) MRE11 protection analysis reveals the importance of RAD51 DNA-binding activity and filament assembly. DNA-binding defective mutants RAD51 Y232W (YW) and T131P (TP) were included in the analysis. BCDX2 at the indicated concentration, RAD51 WT, YW (**C**) or TP (**D**), and MRE11 were incubated with 58 nM Cy3-labeled 5′ overhang DNA substrate at 37°C for 40 min. The left panel is a representative gel image, and the right panel shows quantitative data (mean ± SD) calculated from at least three independent repeats. Statistical significance was determined using one-way ANOVA with Tukey's post hoc test; ns not significant, *P < 0.05, **P < 0.01, ***P < 0.001, ****P < 0.0001.

Considering BCDX2 alone demonstrates only modest protective activity (Supplementary Figures S4A), and given the previous report identifying RAD51 as a *bona fide* fork protector (18,19,23), we hypothesized that RAD51 plays a dominant role in RAD51-BCDX2 collaborative fork protection. To dissect the underlying mechanism, we concentrated on establishing crucial attributes of RAD51’s biochemical activities. RAD51 harbors a Walker A motif involved in ATP binding and hydrolyzes ATP in a DNA-dependent manner (49). We wondered if ATP is required for the DNA protection mediated by RAD51 in conjunction with BCDX2. Indeed, we found that there was a 2-fold increase in fully protected DNA upon the addition of ATP (Figure 3A, comparing lanes 9 with 10), supporting the idea that ATP is necessary for maximal DNA protection.

Next, we delineated how ATP mediates the function of RAD51 in protecting DNA. It is well-known that RAD51 can bind DNA even in the absence of ATP (49). However, in the presence of ATP, RAD51 can form an ATP-bound filament, which facilitates DNA extension, resulting in the DNA structure being extended 1.5-fold relative to classical B-form DNA (49,62). This ATP-bound filament is defined as being a ‘functional’ RAD51 filament. The RAD51 K133A mutant, which lacks ATP binding ability, exhibits DNA binding activity similar to that of WT RAD51 (49), but it is unable to form a ‘functional filament’ due to its inability to bind ATP (49). Given this separation-of-function characteristic, we utilized the RAD51 K133A mutant to examine if ATP binding by RAD51 and subsequent functional filament are required in fork protection (Figure 3B and Supplementary Figure S7A and B). As shown in Figure 3B, the RAD51 K133A mutant protein was impaired in terms of the synergistic action with BCDX2 in DNA protection. This result demonstrates that functional RAD51 filament is a prerequisite for BCDX2-mediated fork protection.

Binding ATP enables the formation of a functional RAD51 filament (49,62). In contrast, hydrolyzing ATP drives the turnover of RAD51 from DNA (49). As a result, blocking ATP hydrolysis will lead to the stabilization of a functional RAD51 filament (49). To gain a comprehensive understanding of the role of RAD51 filament in fork protection, we included the RAD51 K133R mutant in our protection analysis. Consistent with previous studies (23,49), the RAD51 K133R mutant, which is competent in binding but not in hydrolyzing ATP, forms a hyperstable RAD51 filament on DNA and exhibits significantly higher protection activity than wild-type RAD51 (Supplementary Figures S7C, comparing lanes 7 with 9). In addition, BCDX2 has only a marginal effect in enhancing the protective activity of the K133R mutant, suggesting a low dependency of the RAD51 K133R mutant for BCDX2 due to its hyperstability (Supplementary Figures S7C, comparing lanes 7 with 8). Notably, the protective effect of the BCDX2-RAD51 WT ensemble is comparable to that of the RAD51 K133R mutant alone, supporting that BCDX2 exerts a stabilizing influence on RAD51 (Supplementary Figures S7C, comparing lanes 7 with 10).

Together, we have shown that ATP is necessary for the synergistic action of BCDX2-RAD51 in DNA protection, and we have also furnished direct evidence for the relevance of ATP binding by RAD51 in this regard.

### Importance of RAD51 DNA binding and nucleoprotein stability in DNA protection

Next, we investigated the importance of the DNA-binding activity of RAD51 for DNA protection. We expressed and purified the RAD51 Y232W mutant protein (Supplementary Figure S7A and B) that harbors a change of tyrosine 232 to tryptophan in its DNA-binding loop 1, which is known to significantly attenuate the DNA-binding activity of RAD51 (63). Biochemical testing with BCDX2 revealed that RAD51 Y232W proved much less proficient than its wild-type counterpart in DNA protection (Figure 3C, comparing lanes 5 and 9).

Next, we tested the RAD51 T131P mutant protein to further address the importance of a stable RAD51 nucleoprotein filament in DNA protection (Supplementary Figure S7A and B). The RAD51 T131P mutation, which engenders a Fanconi anemia-like phenotype, negatively impacts the stability of RAD51 nucleoprotein filaments (24). Interestingly, DNA fiber analysis revealed that the RAD51 T131P variant is proficient in promoting fork reversal, yet it is defective in fork protection (18,19). With purified RAD51 T131P, our *in vitro* reconstituted assay showed that substituting wild-type RAD51 with T131P variant dramatically attenuates the synergistic protection effect of RAD51-BCDX2 (Figure 3D, comparing lanes 5 and 9), suggesting that a stable filament is required for protecting forks. We also examined the RAD51 II3A mutant, which previous DNA fiber analysis revealed to be competent in fork protection but not in homologous recombination (64). Our results show that RAD51 II3A synergizes with BCDX2 at higher concentrations in fork protection (Supplementary Figures S7D), which aligns with previous research suggesting that the RAD51 II3A mutant protects reversed forks (64).

Together, by using mutants deficient in DNA binding (Y232W and T131P) and a mutant defective in functional filament formation (K133A), our results provide strong evidence that the DNA protection afforded by the BCDX2–RAD51 ensemble is contingent upon the ability of RAD51 to bind DNA and to form a stable nucleoprotein filament.

### Specificity of BCDX2–RAD51 in preventing MRE11 degradation

Next, we examined if enhanced BCDX2-mediated DNA protection is specific to human RAD51 protein. To do so, we tested *E. coli* RecA protein and yeast Rad51 (yRad51) protein, orthologs of human RAD51, with BCDX2 in a MRE11 protection assay. Both RecA and yRad51 only marginally enhanced DNA protection in association with BCDX2 (Supplementary Figure S8A and B), providing the evidence of species specificity for the synergic BCDX2-RAD51 activity in DNA protection. Notably, we observed that the BCDX2-RAD51 ensemble also effectively protects dsDNA from digestion by *E. coli* ExoIII, which, like MRE11, exerts exonuclease activity with a 3′-to-5′ polarity and serves as an excellent control in our mechanistic study (Supplementary Figure S8C). Taken together, our results indicate that the DNA protective effect extends beyond the human MRE11 nuclease despite the species selectivity of BCDX2–RAD51 synergy.

### BCDX2 synergizes with RAD51 to protect DNA from EXO1 nucleolytic degradation

**Figure 4.**
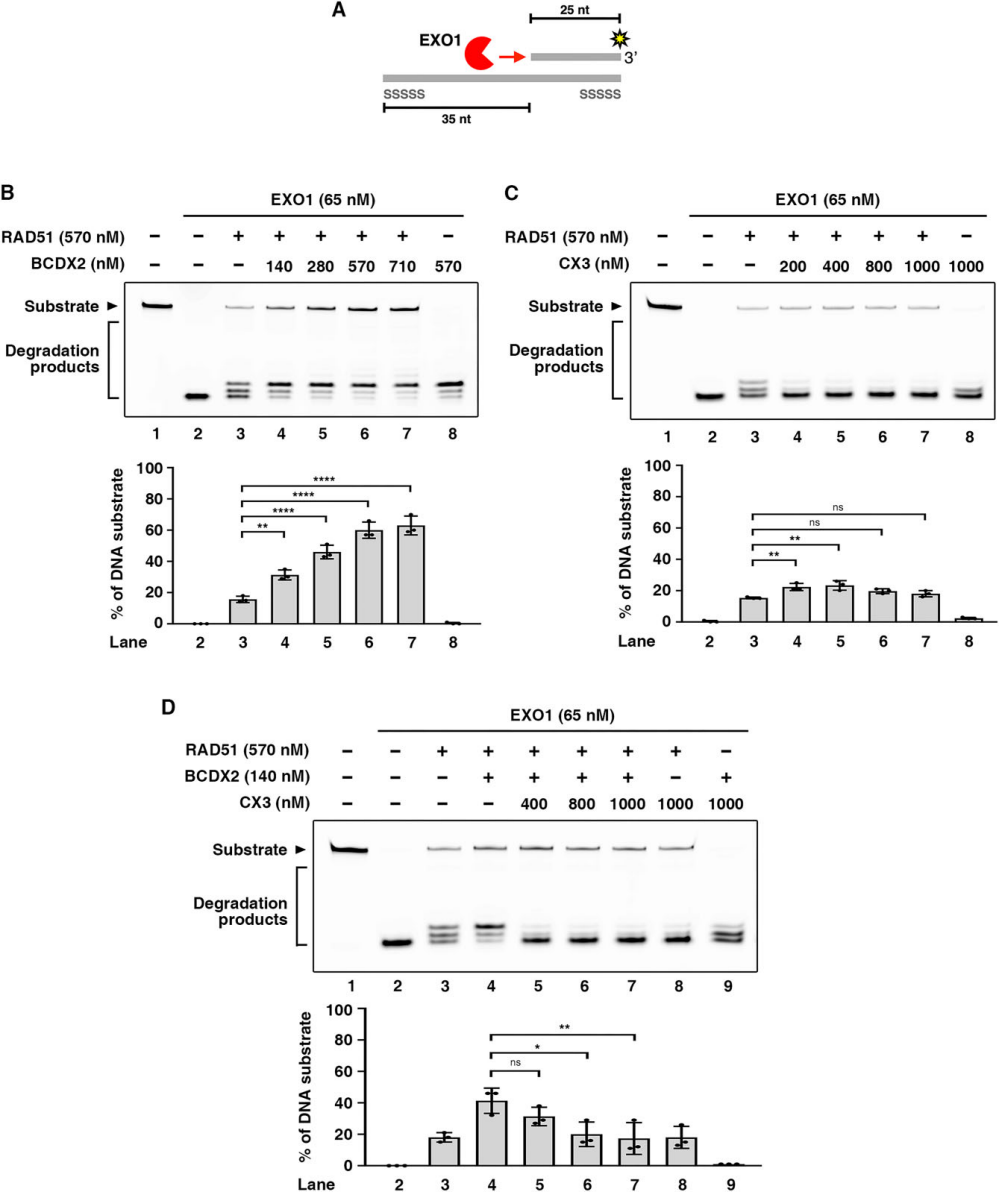
BCDX2 and RAD51 synergistically protect DNA against EXO1 nucleolytic degradation. (**A**) Illustration of the EXO1 protection assay with a 3′ overhang DNA substrate designed to mimic the regression arm of a reversed fork. The 3′ overhang DNA substrate is composed of a 3′ Cy3-labeled 25 nt oligonucleotide and a 60 nt oligonucleotide with a phosphorothioate bond modification on both ends to avoid nonspecific nucleolytic degradation. (**B** and **C**) EXO1 protection analysis. BCDX2 (**B**) or CX3 (**C**) at the indicated concentration, RAD51, and EXO1 were incubated with 58 nM Cy3-labeled 3′ overhang DNA substrate at 37°C for 40 min. The reactions were then terminated and resolved in a 27% denatured polyacrylamide gel. (**D**) The indicated concentrations of BCDX2, CX3, RAD51 and EXO1 were incubated with Cy3-labeled 3′ overhang DNA substrate as described above. For (B–D), the top panel is a representative gel image, and the bottom panel shows quantitative data (mean ± SD) calculated from at least three independent repeats. Statistical significance was determined using one-way ANOVA with Tukey's post hoc test; ns not significant, *P < 0.05, **P < 0.01, ****P < 0.0001.

The above-described findings prompted us to consider if the BCDX2-RAD51 ensemble may exert negative regulation over multiple cellular nucleases, such as the 5′-to-3′ exonuclease EXO1 that is involved in pathological fork degradation upon replication stress (12,14,15). Accordingly, we examined if BCDX2 synergizes with RAD51 to protect dsDNA against the action of EXO1. To do so, human EXO1 was expressed in human cells and purified to a high degree of homogeneity (Supplementary Figure S9A). Purified EXO1 was tested in a reconstituted DNA protection assay with DNA substrate harboring a 3′ overhang (Figure 4A and Supplementary Figure S9B). We found that BCDX2 and CX3 complexes alone could barely protect DNA from EXO1 activity (Supplementary Figure S9C and D). Importantly, BCDX2 significantly synergized with RAD51 in protecting DNA from EXO1 degradation (Figure 4B), and marginal protection was detected for CX3-RAD51 (Figure 4C). Finally, the inclusion of CX3 did not further enhance the BCDX2-RAD51-mediated protective effect (Figure 4D). Thus, our results reveal that BCDX2 and CX3 confer varying degrees of protection and that BCDX2 significantly cooperates with RAD51 to protect DNA from attack by nucleases other than MRE11.

### Impairment of BCDX2–RAD51-mediated DNA protection by cancer-associated RAD51C mutations

**Figure 5.**
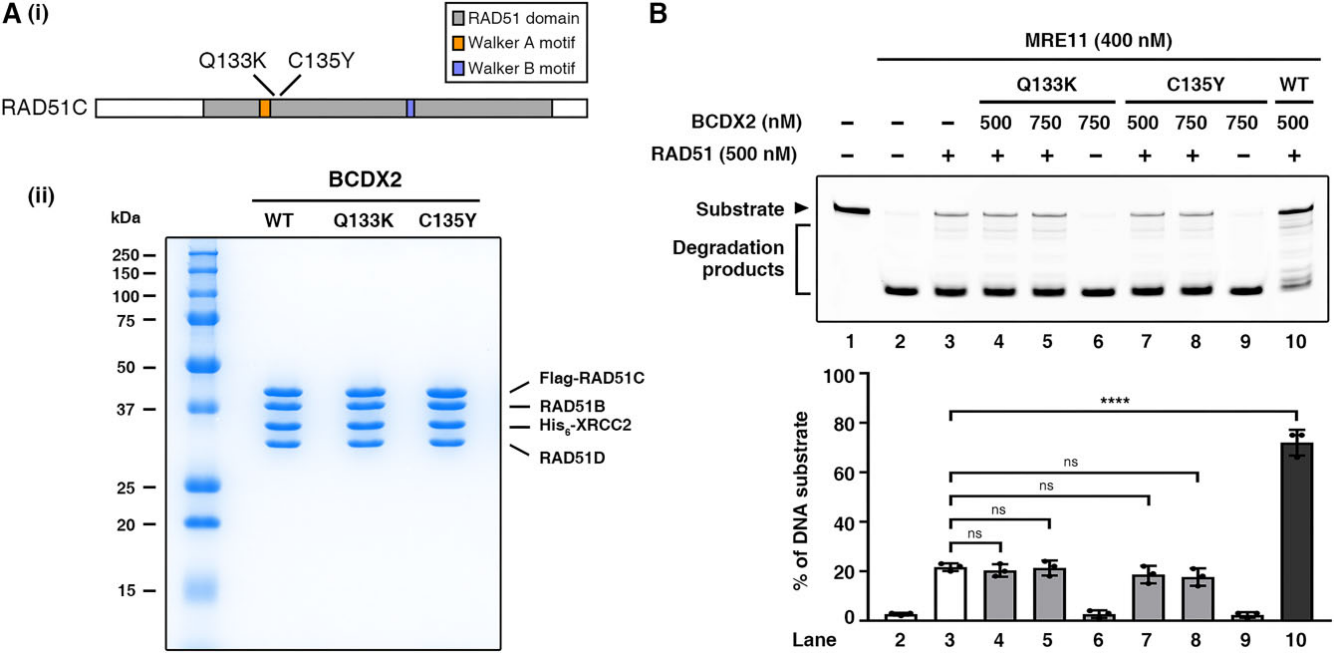
Cancer-associated mutant variants of RAD51C are defective in collaborative fork protection. (**A**) (i) Schematic showing the Q133K and C135Y mutation sites adjacent to the Walker A motif of RAD51C. (ii) The purified BCDX2 Q133K and C135Y mutant complexes (2.5 μg protein for each lane) were analyzed in a 12% SDS-denaturing polyacrylamide gel with Coomassie Blue staining. (**B**) MRE11 protection assay. BCDX2 (WT, Q133K or C135Y) at the indicated concentration, RAD51, and MRE11 were incubated with 58 nM Cy3-labeled 5′ overhang DNA substrate at 37°C for 40 min. The reactions were then terminated and resolved in a 27% denatured polyacrylamide gel. The top panel is a representative gel image, and the bottom panel shows quantitative data (mean ± SD) calculated from at least three independent repeats. Statistical significance was determined using one-way ANOVA with Tukey's post hoc test; ns not significant, ****P < 0.0001.

In a recent study, Prakash *et al.* conducted a comprehensive analysis to reveal the functional impact of various RAD51C missense mutations found in breast and ovarian cancer patients (51). Two such RAD51C mutant variants, Q133K and C135Y, display impaired HR activity, but their role in replication fork protection remains to be determined. Therefore, we examined how these RAD51C mutations might affect the ability of BCDX2 to function with RAD51 in protecting DNA from nuclease attacks. BCDX2 complexes with either the RAD51C Q133K or C135Y mutations were expressed in human cells and purified using procedures developed for the wild-type protein complex (Figure 5A). Importantly, our DNA protection analysis revealed that neither of the two mutant BCDX2 complexes was functional (Figure 5B). Thus, our results provide biochemical support for the premise that the aforementioned RAD51C cancer-associated mutations affect replication fork protection.

## Discussion

RAD51 paralogs are indispensable for cell survival and genome stability (34–37,51,65,66). Apart from their canonical role in homology-directed repair, RAD51 paralogs have been implicated in the restart of stalled replication forks (44–46). Using highly-purified and monodispersed RAD51 paralog protein complexes, we have conducted experiments to define the role of BCDX2 and CX3 in regulating reversed fork dynamics. We have shown that BCDX2 and CX3 lack replication fork reversal activity alone or in combination with RAD51. Interestingly, whereas BCDX2 and CX3 can barely protect reversed forks, BCDX2 exhibits a significant synergistic effect with human RAD51 in protecting DNA from MRE11- and EXO1-mediated nucleolytic degradation. Though this BCDX2 activity is specific for human RAD51, DNA protection occurs even when these proteins were tested against a heterologous exonuclease. Importantly, the DNA-binding activity and a stable nucleoprotein filament of RAD51 are both important for the observed functional synergy with BCDX2 in protecting DNA. Moreover, we have shown that clinically relevant mutations in RAD51 and BCDX2 impair their ability to protect DNA, providing a possible explanation for the clinical impact of such mutations.

A previous cell-based electron microscopy analysis evidenced a significant decrease in the number of reversed forks in RAD51C- or RAD51D-depleted cells (46), indicating that RAD51 paralogs are involved in reversed fork formation. Surprisingly, our biochemical analysis shows that neither BCDX2 nor CX3 mediates replication fork reversal with or without RAD51, indicating that: (i) the RAD51 paralogs, even in conjunction with RAD51, lack fork reversal activity and (ii) additional essential factors are required in the process. More specifically, it is likely that the RAD51 paralogs augment the activity of DNA motor proteins capable of catalyzing fork reversal. Consistent with this notion, a recent study has revealed that BCDX2, along with RAD51, can facilitate motor-driven strand annealing activity, likely representing one of the initial activities involved in promoting fork reversal by the fork remodelers SMARCAL1 and ZRANB3 (56). The various functional interactions of such fork remodelers with RAD51 and the RAD51 paralogs in terms of how they promote the formation of reversed forks warrants further exploration.

Protection of reversed forks is a critical aspect of restarting stalled forks. Herein, our results demonstrate a synergistic effect of BCDX2 and RAD51 in protecting DNA against MRE11 and EXO1 nuclease degradation. Our study is also consistent with the notion that significant degradation of nascent DNA may be observed by knocking down RAD51 paralogs in a DNA fiber analysis (45). Paradoxically, a recent study by Berti *et al.* indicated that the integrity of stalled replication forks is not compromised by knocking out RAD51C, RAD51D or XRCC3 in U2OS cells (46). Interestingly, similar paradoxes have also been reported for RAD51 knockdown cells, with different knockdown levels of RAD51 resulting in two opposing phenotypic consequences, i.e. fork preservation or degradation (54). These conflicting results can be rationalized by the different amounts of RAD51 required in the two sequential steps, with less RAD51 being sufficient for fork reversal, whereas more is required for fork protection (54). Extending that notion, we hypothesize that a similar scenario could be applied to RAD51 paralogs since (i) RAD51 paralogs are also perquisites for both fork reversal and protection, and (ii) different degrees of fork degradation are also observed in knockdown (KD) and knockout (KO) cells of RAD51 paralogs (45,46). The hypothesis that fork reversal and protection may require different cellular amounts of RAD51 paralogs explains the aforementioned conflicting results and needs further elucidation.

Our study demonstrates that BCDX2 and RAD51 exhibit synergistic DNA protection against various nucleases, including *E. coli* ExoIII, indicating that BCDX2 regulates a fundamental attribute of RAD51-DNA nucleoprotein filaments essential for DNA protection. We propose that BCDX2 may modulate RAD51 assembly on DNA to form a non-specific roadblock against various nucleases. The broad protection effect is consistent with a study by Halder *et al.* showing that the DNA protective effect of RAD51 also extends beyond specific nucleases (23). In addition, given that the RAD51 paralogs are conserved RAD51 modulators and can significantly affect the number of cellular RAD51 foci (34,40,52,53), it is tempting to speculate that BCDX2 enhances RAD51’s protective activity by affecting a fundamental attribute of RAD51–DNA nucleoprotein filaments. Single-molecule experiments in *Caenorhabditis elegans* have revealed that the RAD-51 paralogs RFS-1/RIP-1 enhance the extension of RAD-51 filament in a 3′-to-5′ direction by transiently binding on the 5′ end of the RAD-51 filament (67). Single-molecule DNA curtain experiments on a yeast complex of two Rad51 paralogs, i.e. Rad55–Rad57, have yielded evidence that it promotes Rad51 filament growth on ssDNA via transient interactions (68). Moreover, recent studies on human RAD51 paralogs using single-molecule and electron microscopy approaches have also demonstrated BCDX2’s ability to enhance RAD51 filament nucleation and extension while not maintaining a stable association with the RAD51–DNA complex (61,69). These findings support the premise that human BCDX2 affects the dynamics of RAD51–DNA nucleoprotein filaments. Consistent with this hypothesis, our results have demonstrated a species-specific functional interaction between human BCDX2 and RAD51. In addition, our protection analysis unveiled the deficiency of the two cancer-associated mutants, BCDX2 Q133K and C135Y, in RAD51-mediated fork protection. Interestingly, Prakash *et al.* reported lower and defective DNA binding activity for these mutants compared to wild-type BCDX2, respectively (51). The results indicate that the activity of BCDX2 in RAD51-mediated fork protection is likely contingent upon its DNA binding ability. Our findings also align well with a single-molecule study supporting a crucial requirement of DNA binding for BCDX2 to promote RAD51 filament growth (61). Further studies are needed to delineate the detailed mechanism by which BCDX2 regulates the dynamics of RAD51-DNA nucleoprotein assembly and/or maintenance.

A myriad of mutations in RAD51 paralogs have been associated with Fanconi anemia and various types of cancer (35–37,51,65,66). Defining the functional impact of these mutations will shed light on their possible pathogenicity and be valuable to patient stratifications for cancer treatment and care. Accordingly, our protein purification protocols and reconstituted systems of DNA protection should prove valuable for defining how mutations in the BCDX2 complex affect its role in protecting stressed replication forks from nucleolytic attrition. Our findings reveal the functional role of RAD51 paralogs in fork protection. In addition, given that RAD51 paralogs also participate in DSB repair, our purified proteins and the biochemical system we have developed can provide a foundation for future studies on the essential functions *in vivo* of paralogs involved in both replication stress and DSB repair.

With regard to the CX3 complex, our biochemical assay demonstrates that while BCDX2 is the major component involved in fork protection, CX3 also contributes to RAD51-mediated fork protection. This observation aligns with a cell-based DNA fiber analysis suggesting that the CX3 complex maintains the integrity of stalled replication forks (44,45). Apart from its role in fork protection, previous reports have also indicated that the CX3 complex is crucial in restarting stalled replication forks, i.e. downstream of the reversed fork formation and protection processes (44–46). Thus, the multiple functional roles of CX3 in replication stress require further investigation.

## Supplementary Material

gkad856_Supplemental_File

## Data Availability

The relevant data are described in the Supplementary Information. Other additional data that support the findings of this study are available from the authors upon reasonable request.
