# Supplementary material for: RAD51 paralogs synergize with RAD51 to protect reversed forks from cellular nucleases
Source: Nucleic Acids Res. 2023 Oct 16;51(21):11717–31. doi: 10.1093/nar/gkad856 (PMC10681713; doi:10.1093/nar/gkad856)
Supplement: gkad856_Supplemental_File [file gkad856_supplemental_file.pdf]

Supplementary Information

RAD51 paralogs synergize with RAD51 to protect reversed forks from cellular nucleases

Chia-Lun Guh[1], Kai-Hang Lei[1], Yi-An Chen[2], Yi-Zhen Jiang[1], Hao-Yen Chang[1,3], Hungjiun Liaw[4], Hung-Wen Li[3], Hsin-Yung Yen[1,2], and Peter Chi[1,2*]

[1]Institute of Biochemical Sciences, National Taiwan University, Taipei, Taiwan
[2]Institute of Biological Chemistry, Academia Sinica, Taipei, Taiwan
[3]Department of Chemistry, National Taiwan University, Taipei, Taiwan
[4]Department of Life Sciences, National Cheng Kung University, Tainan City, Taiwan

[*] To whom correspondence should be addressed:
Peter (Hung Yuan) Chi
Address:
Institute of Biochemical Sciences
College of Life Science
National Taiwan University
NO. 1, Sec. 4, Roosevelt Rd., Taipei, 10617 Taiwan
Email: peterhchi@ntu.edu.tw
Phone: 886-2-23665573
Fax: 886-2-23635038

This PDF file includes:
Supplementary Tables S1 to S3
Supplementary Figures S1 to S9

Supplementary Table S1. Protein identification of BCDX2 and CX3 complexes by liquid chromatography-tandem mass spectrometry (LC-MS/MS). The identity of BCDX2 and CX3 complexes is confirmed by liquid chromatography-tandem mass spectrometry (LC-MS/MS).

	Subunits	Coverage (%)	Number of unique peptides
**BCDX2**	RAD51B	28	12
	RAD51C	46	13
	RAD51D	63	15
	XRCC2	56	13
**CX3**	RAD51C	69	42
	XRCC3	82	39

Supplementary Table S2. Mass assignment and stoichiometry determination for complexes and sub-complexes analyzed by native mass spectrometry.

Measured Mass ± SD (Da)	Complex/Sub-complex	Theoretical Mass (Da)	ΔMass (Da)	% Mass Error
153,474.3 ± 79.6	RAD51B+ Flag-TEV-RAD51C+ RAD51D+His-TEV-XRCC2	151,236.4	2,237.9	1.47
68,719.0 ± 0.1	RAD51D+His-TEV-XRCC2	68,811.7	-92.7	-0.13
82,370.5 ± 1.2	RAD51B+Flag-TEV-RAD51C	82,424.7	-54.2	-0.06
38,164.2 ± 0.7	RAD51B	38,257.0	-92.8	-0.24
44,207.9 ± 1.4	Flag-TEV-RAD51C	44,167.7	40.2	0.09
34,917.2 ± 0.7	RAD51D	35,049.3	-132.1	-0.37
33,801.8 ± 0.5	His-TEV-XRCC2	33,762.4	39.4	0.11

Supplementary Table S3. List of oligonucleotides used in this study. Cy3: represents Cy3 fluorescent dye-labelled on the 5'- or 3'- end of the indicated oligonucleotide. The asterisks represent phosphorothioate bond modification between two nucleotides.

Name	DNA sequence (5' to 3')
Oligo 1	Cy3-CGTGACTTGATGTTAACCCTAACCCTAAGATATCGCGTTATCAGAGTGTGAGGATACATGTAGGCAATTGCCACGTGTCTATCAGCTGAAGTTGTTCGCGACGTGCGATCGTCGCTGCGACG
Oligo 2	CGTCGCAGCGACGATCGCACGTCGCGAACAACTTCAGCTGATAGACACGTGG
Oligo 3	Cy3-TCAGAGTGTGAGGATACATGTAGGCAATTGCCACGTGTCTATCAGCTGAAGTTGTTCGCGACGTGCGATCGTCGCTGCGACG
Oligo 4	CGTCGCAGCGACGATCGCACGTCGCGAACAACTTCAGCTGATAGACACGTGGCAATTGCCTACATGTATCCTCACACTCTGAATACGCGATATCTTAGGGTTAGGGTTAACATCAAGTCACG
Oligo 5	CGTCGCAGCGACGATCGCACGTCGCGAACAACTTCAGCTGATAGACACGTGGCAATTGCCTACATGTATCCTCACACTCTGA
Oligo 6	Cy3-CCACGTGTCTATCAGCTGAAGTTGTTCGCGACGTGCGATCGTCGCTGCGACG
Oligo 7	Cy3-GGGTGAACCTGCAGGTGGGCAAAGA
Oligo 8	A*C*G*C*T*GCCGAATTCTACCAGTGCCTTGCTAGGACATCTTTGCCCACCTGCAGGTT*C*A*C*C*C
Oligo 9	AGGCACTGGTAGAATTCGGCAGCGT-Cy3
Oligo 10	T*T*T*A*G*CTGCATATTTACAACATAGCGCAATACAGCACCAGATTCAGCAATTAAGC*T*C*T*A*A
Oligo 11	T*A*A*T*A*CAAAATAAGTAAATGAATAAACAGAATTGCGCTATGTTGTAAATATGCAG*C*T*A*A*A
Oligo 12	A*C*G*C*T*GCCGAATTCTACCAGTGCCTTGCTAGGACATCTTTGCCCACCTGCAGGTTCACCCTCTGTTTATTCATTTACTTATTTTG*T*A*T*T*A
Oligo 13	Cy3-TTAGAGCTTAATTGCTGAATCTGGTGCTGTGGGTGAACCTGCAGGTGGGCAAAGA
Oligo 14	TCTTTGCCCACCTGCAGGTTCACCCACAGCACCAGATTCAGCAATTAAGCTCTAA

Supplementary Figure S1

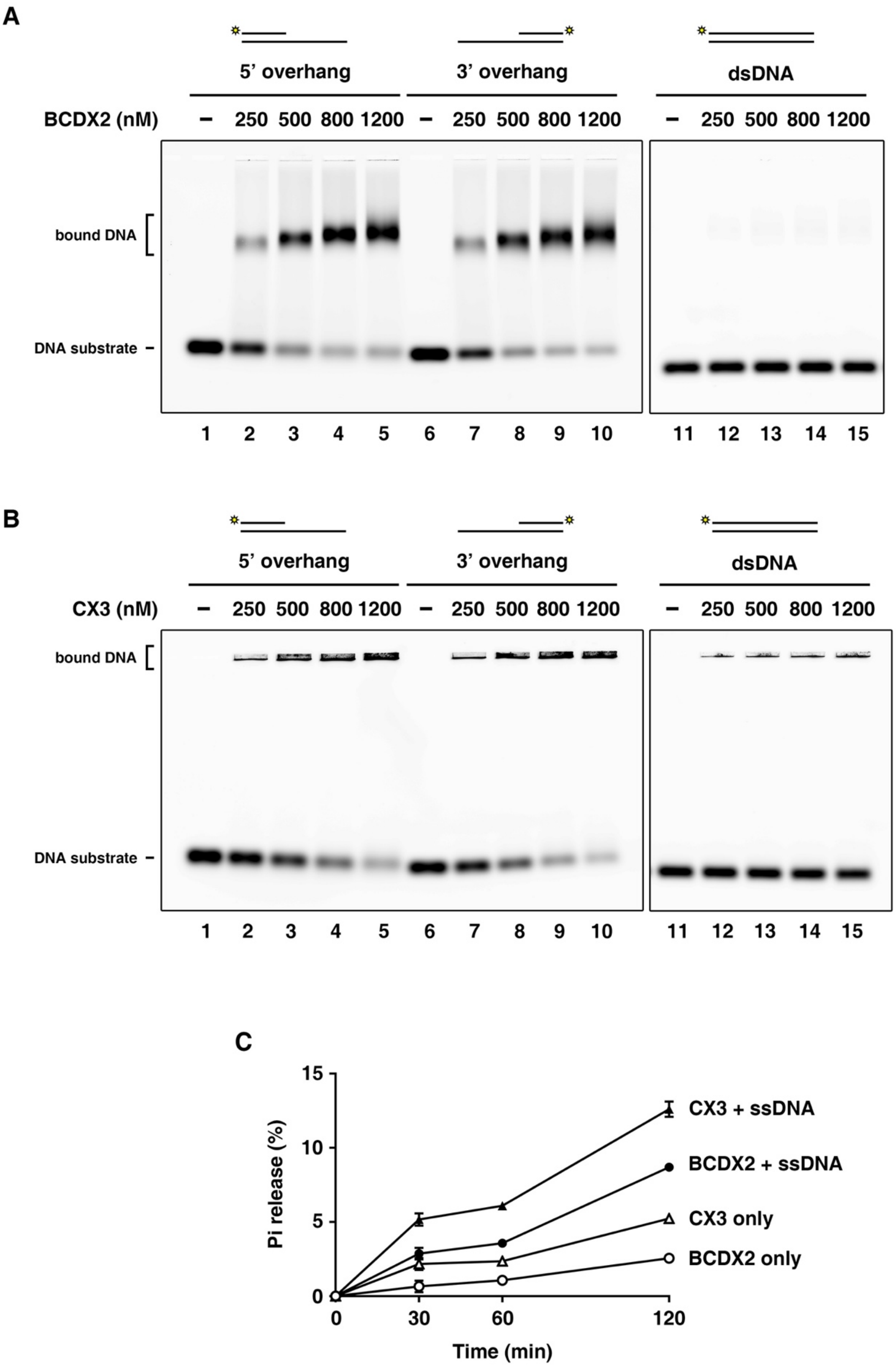

Supplementary Figure S1. Purified BCDX2 and CX3 are competent in DNA binding and ATP hydrolysis. (A) DNA binding activity of the BCDX2 complex. BCDX2 binds 5' and 3' overhang DNA substrates but not double-stranded DNA. The indicated DNA substrate (58 nM) was incubated with the indicated concentrations of BCDX2 at 37 °C for 10 min. Samples were analyzed using a 0.8 % agarose gel. **(B)** DNA binding activity of the CX3 complex. CX3 exhibits significant DNA binding activity on 5' and 3' overhang DNA substrates and weak activity on double-stranded DNA. The indicated DNA substrate (58 nM) was incubated with the indicated concentrations of CX3 at 37 °C for 10 min. **(C)** ATPase activity of BCDX2 and CX3. BCDX2 or CX3 (1.58 μM) was incubated with [γ-^{32}P]ATP in the presence or absence of single-stranded DNA (15.8 μM nucleotides, phiX174 virion) for the indicated times. The graph shows quantitative data (mean ± SD) calculated from at least three independent repeats.

Supplementary Figure S2

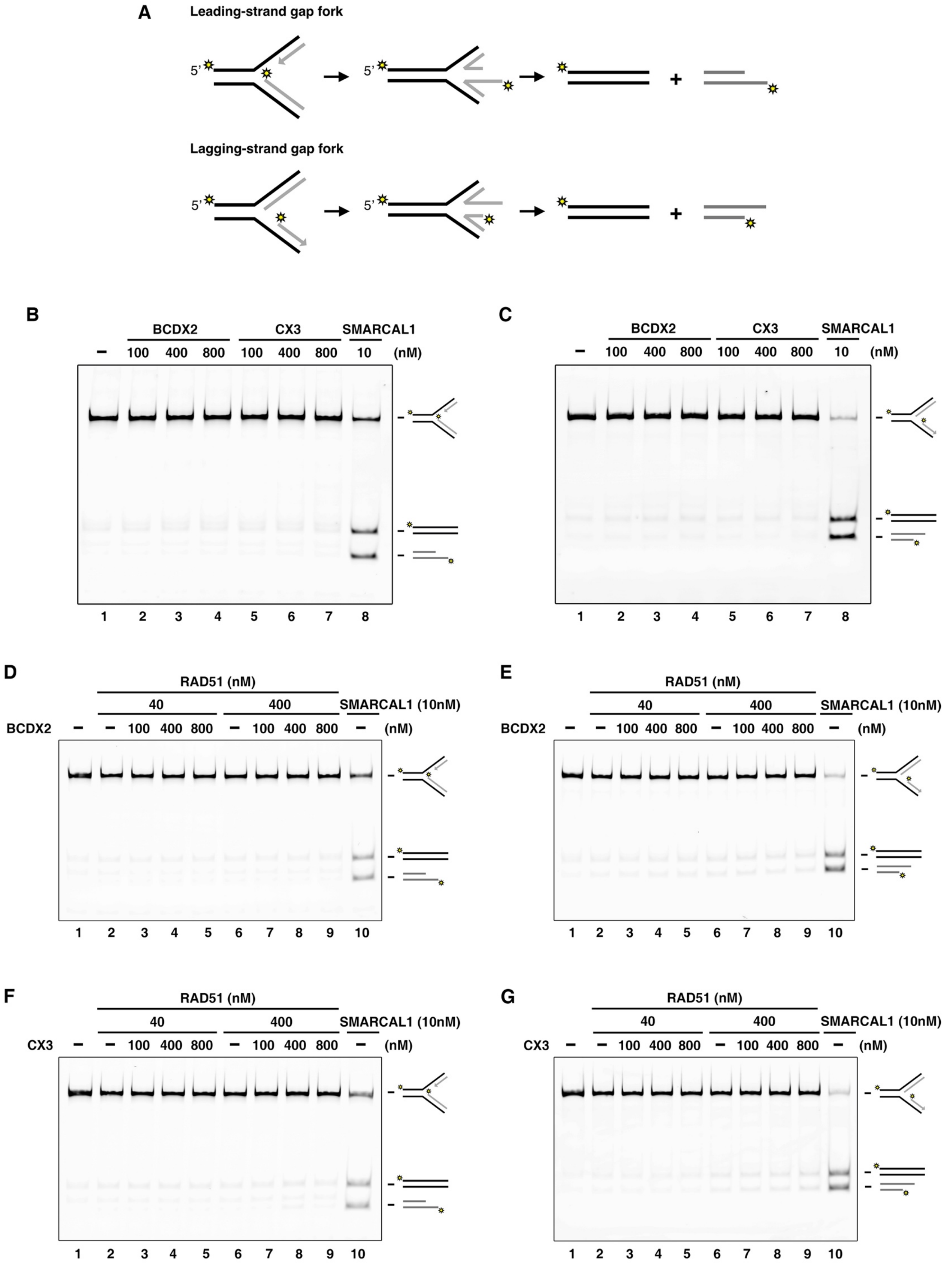

Supplementary Figure S2. BCDX2 and CX3 lack intrinsic fork reversal activity. **(A)** Schematics of fork reversal assay for leading-strand or lagging-strand gap substrates. The asterisk represents the Cy3 fluorescence label on the 5' end of the indicated oligonucleotide. **(B and C)** Fork reversal analysis. The indicated concentration of BCDX2 or CX3 complex was incubated with a leading-strand (B) or lagging-strand (C) gap fork (8 nM in molecules) at 37 °C for 15 min. Reactions were then terminated and resolved in 6 % TBE polyacrylamide gels. **(D and E)** The indicated amount of RAD51 and BCDX2 complex was incubated with a leading-strand (D) or lagging-strand (E) gap fork (8 nM in molecules) at 37 °C for 15 min, and the reactions were stopped and resolved as described above. **(F and G)** The indicated amount of RAD51 and CX3 complex was incubated with a leading-strand (F) or lagging-strand (G) gap fork (8 nM in molecules) at 37 °C for 15 min. The reactions were then terminated and resolved as described above.

Supplementary Figure S3

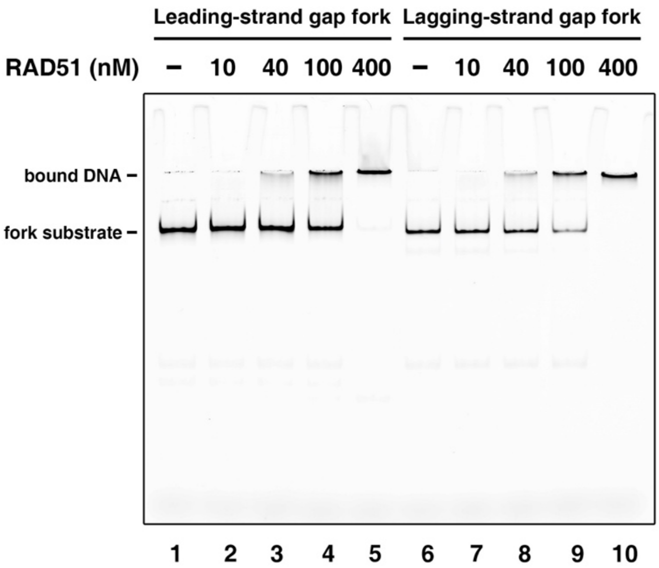

Supplementary Figure S3. Electrophoretic mobility shift assay shows RAD51 shifting different amounts of fork substrates. The indicated amount of RAD51 was incubated with a leading-strand or lagging-strand gap fork (8 nM in molecules for each) at 37 °C for 10 min. Reactions were then resolved in a 6 % TBE polyacrylamide gel.

Supplementary Figure S4

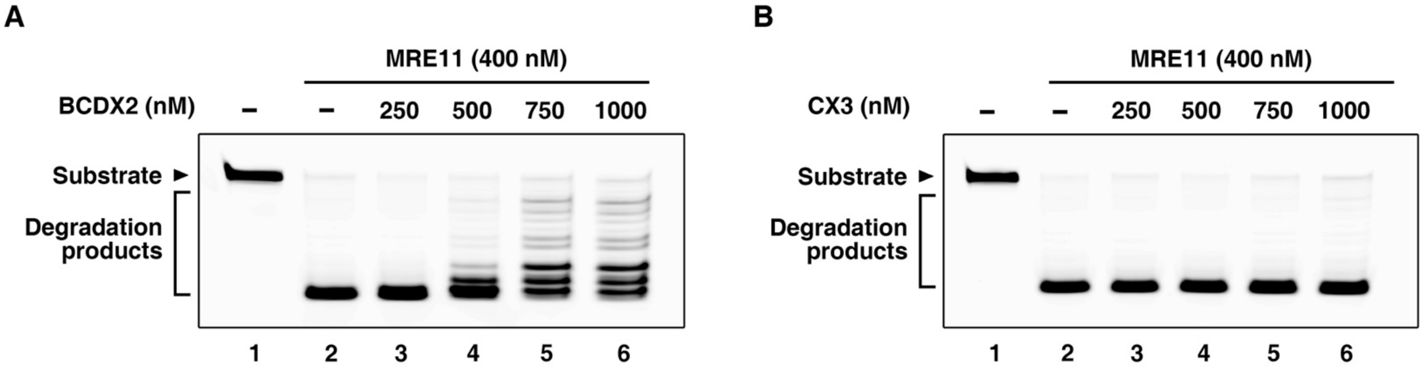

Supplementary Figure S4. BCDX2 and CX3 lack a significant fork protection activity against MRE11. (A and B) MRE11 protection assay. The indicated concentration of BCDX2 (A) or CX3 (B) and MRE11 was incubated with 58 nM Cy3-labeled 5' overhang DNA substrates at 37 °C for 40 min. Reactions were then stopped and resolved in 27% denatured polyacrylamide gels.

Supplementary Figure S5

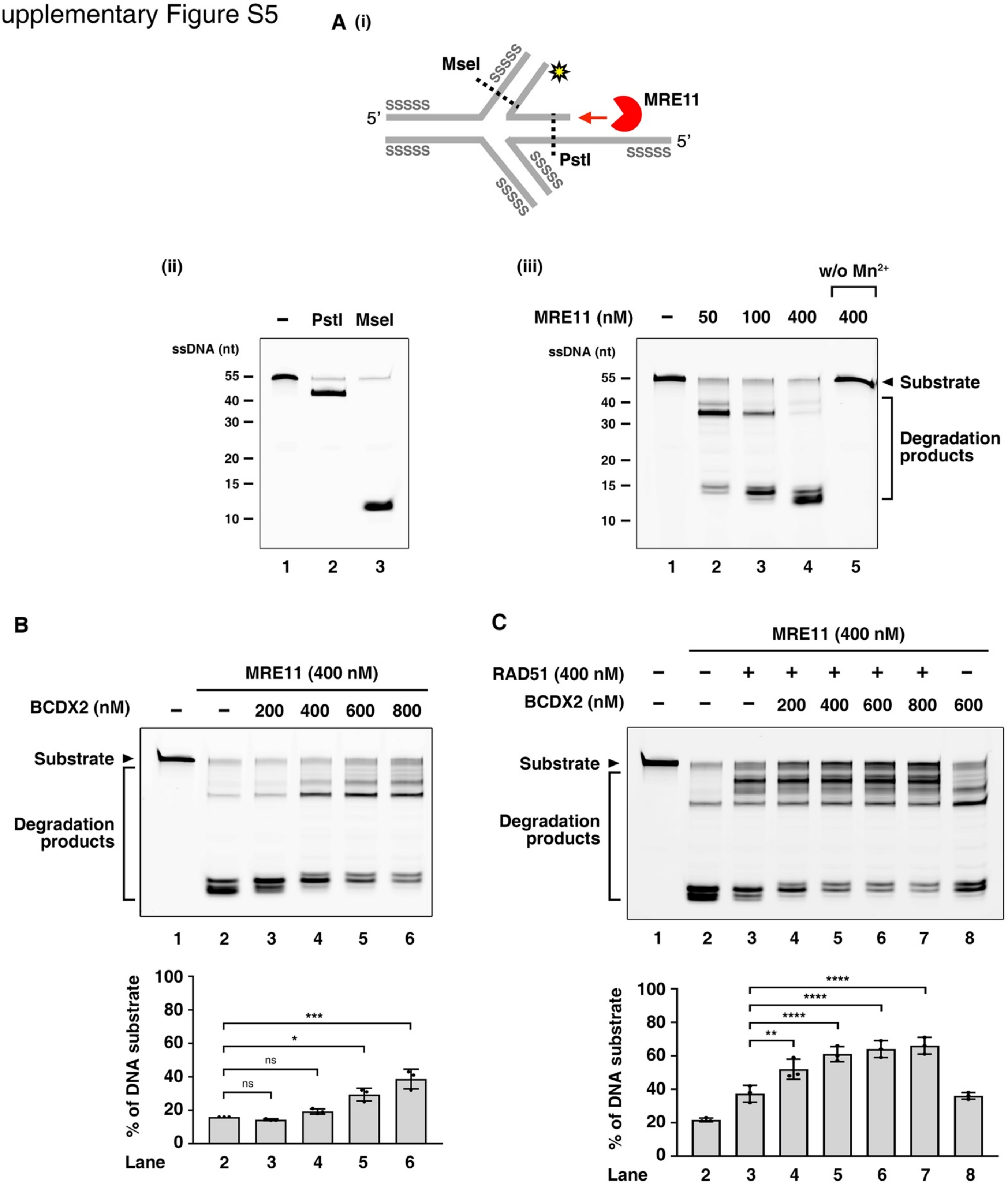

Supplementary Figure S5. BCDX2 enhances the fork protection activity of RAD51 on reversed fork-like DNA substrate. (A) (i) Illustration of a DNA substrate mimicking the structure of a reversed fork. Except for the 5' Cy3-labeled 55 nt oligonucleotide, both ends of other oligonucleotides were modified with a phosphorothioate bond to avoid nonspecific nucleolytic degradation. (ii) The conformation of the designed substrate was verified by restriction enzyme mapping. The cut sites of PstI and MseI restriction enzymes on the DNA substrate are indicated in (i). As expected, DNA digestion by PstI and MseI generates a Cy3-43 nt and a Cy3-8 nt oligonucleotide, respectively. The DNA species are resolved in a 27% denatured gel. (iii) MRE11 protection assay. The indicated concentration of MRE11 was incubated with the reversed fork-like substrate (29 nM) with or without the Mn^{2+} cofactor at 37 °C for 80 min, and the reactions were stopped and resolved as described above. Note that 2.5 mM $MnCl_2$ was used in the reaction buffer to enhance MRE11 activity. **(B and C)** The indicated concentration of BCDX2 alone (B) or BCDX2 with RAD51 (C) was incubated with MRE11 and the reversed fork-like substrate (29 nM) at 37 °C for 80 min. Reactions were terminated and resolved as described above.

B and C: the top panel is the representative gel image; the bottom panel is the quantitative data calculated from at least three independent repeats showing mean ± SD. Statistical significance was determined using one-way ANOVA with Tukey's post hoc test; ns not significant, *P < 0.05, **P < 0.01, ***P < 0.001, ****P < 0.0001.

Supplementary Figure S6

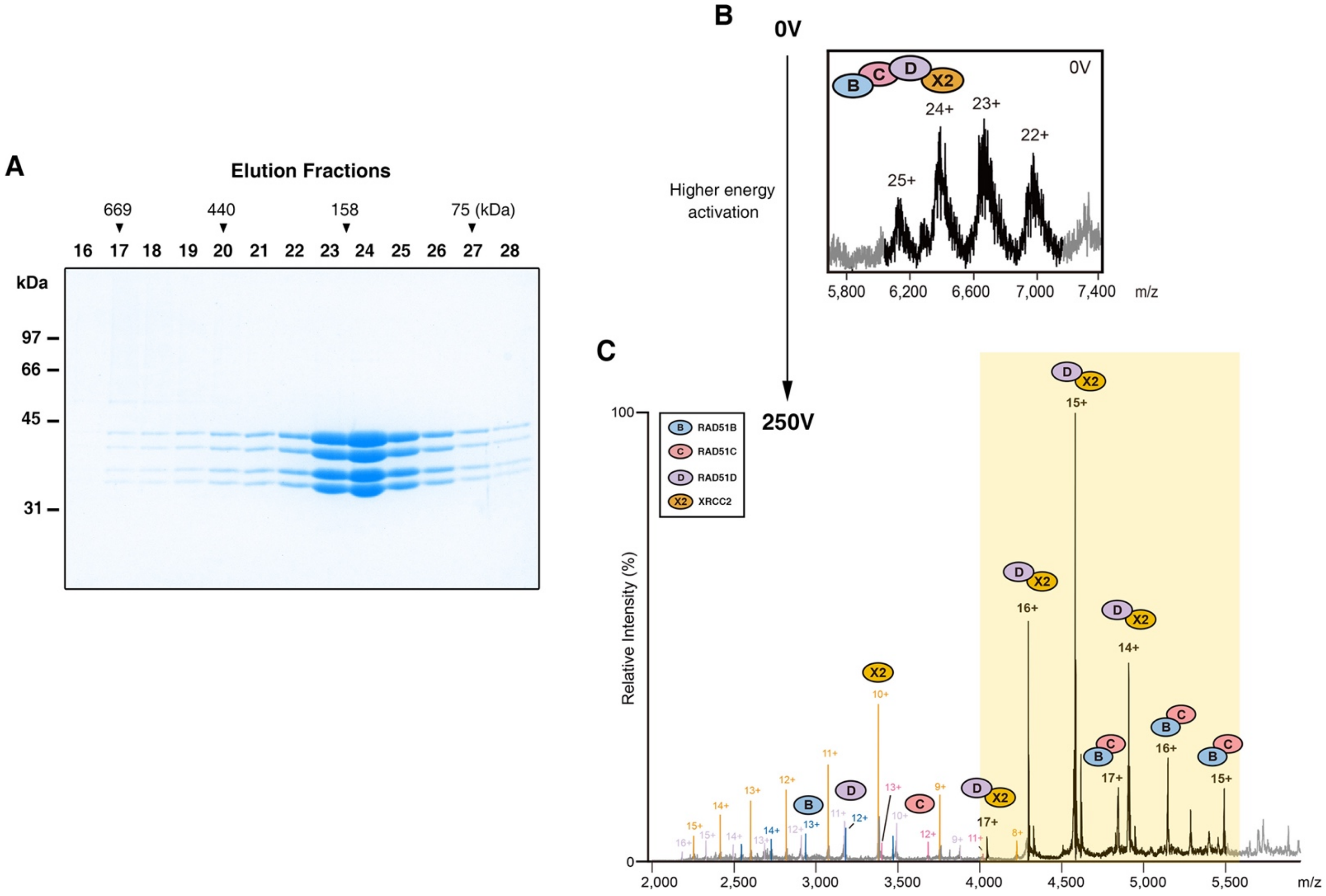

Supplementary Figure S6. Size-exclusion analysis and native mass spectrometry reveal the stoichiometry and composition of the BCDX2 complex. (A) Size-exclusion analysis in Superdex 200 increase column reveals the monomeric status of the BCDX2 complex. Fractions were resolved in a 12% SDS-PAGE gel, visualized with Coomassie blue staining. **(B)** A representative native MS spectrum of the purified BCDX2 complex without in-source activation (0V, black box). The complex stoichiometry was highlighted by illustration. **(C)** A representative MS spectrum of the purified BCDX2 complex under the high in-source trapping energy (250V). The sub-complexes corresponding to RAD51B-RAD51C and RAD51D-XRCC2 were observed, highlighted in the yellow background, to further confirm the stoichiometry of the BCDX2 complex. The signals corresponding to RAD51B, RAD51C, RAD51D, and XRCC2 monomers were colored in blue, pink, purple, and orange with individual charge states, respectively. The number of repeated experiments (n=3).

Supplementary Figure S7

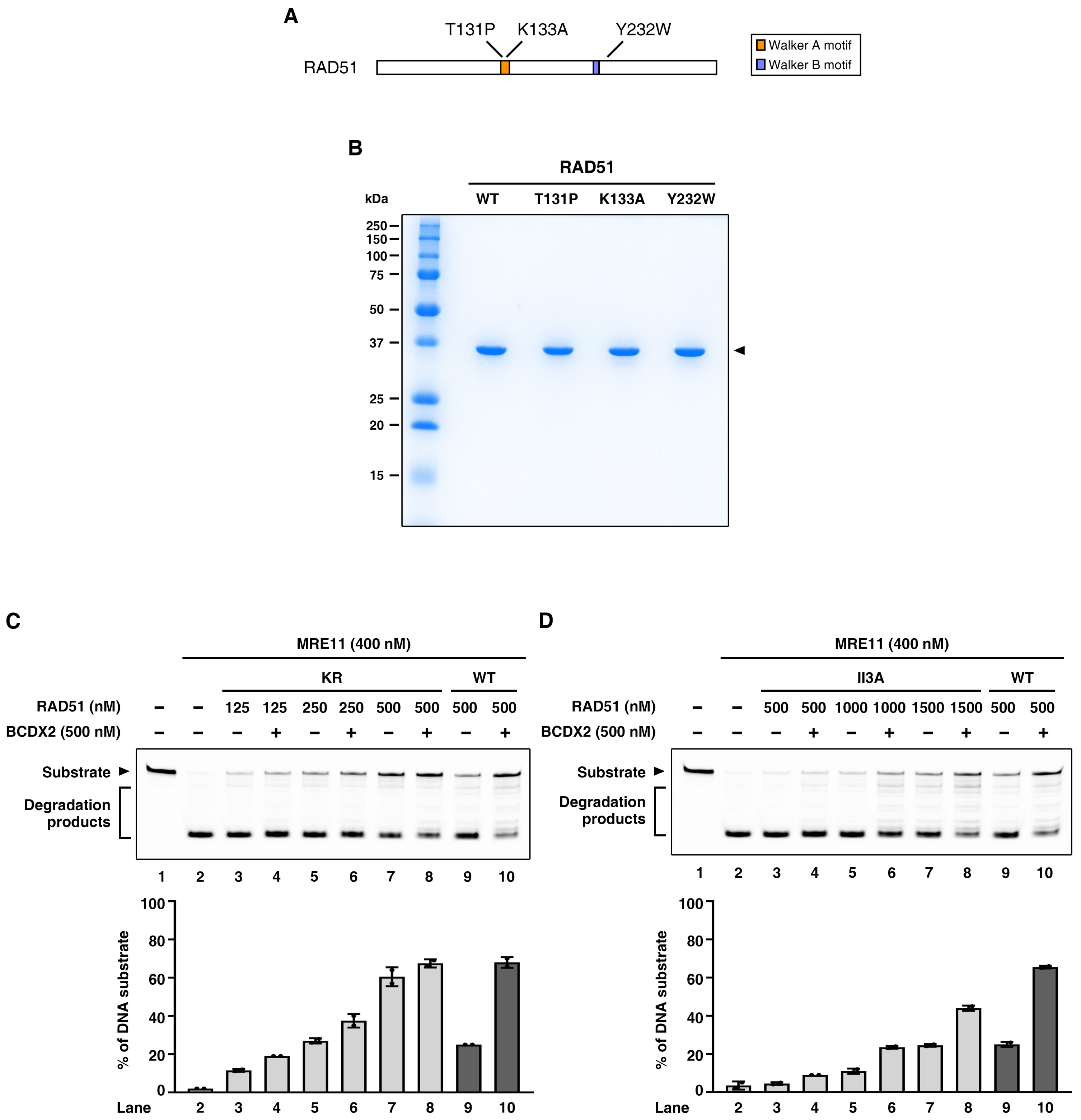

Supplementary Figure S7. Purified RAD51 mutant variants. (A) A scheme of the T131P, K133A, and Y232W mutation sites in RAD51. **(B)** The purified RAD51 T131P, K133A, and Y232W mutant variants (1 μg for each) were analyzed in a 12% SDS-denaturing polyacrylamide gel with Coomassie Blue staining. **(C and D)** MRE11 protection assay in the presence of the ATP-hydrolysis-defective RAD51 K133R (KR) mutant (C) and the strand-exchange-defective RAD51 II3A (II3A) mutant (D). BCDX2, RAD51 wild-type (WT), KR (C), or II3A (D), and MRE11 were incubated with 58 nM Cy3-labeled 5' overhang DNA substrate at 37 °C for 40 min.
For **C and D**, the top panel is a representative gel image, and the bottom panel shows quantitative data (mean ± SD).

Supplementary Figure S8

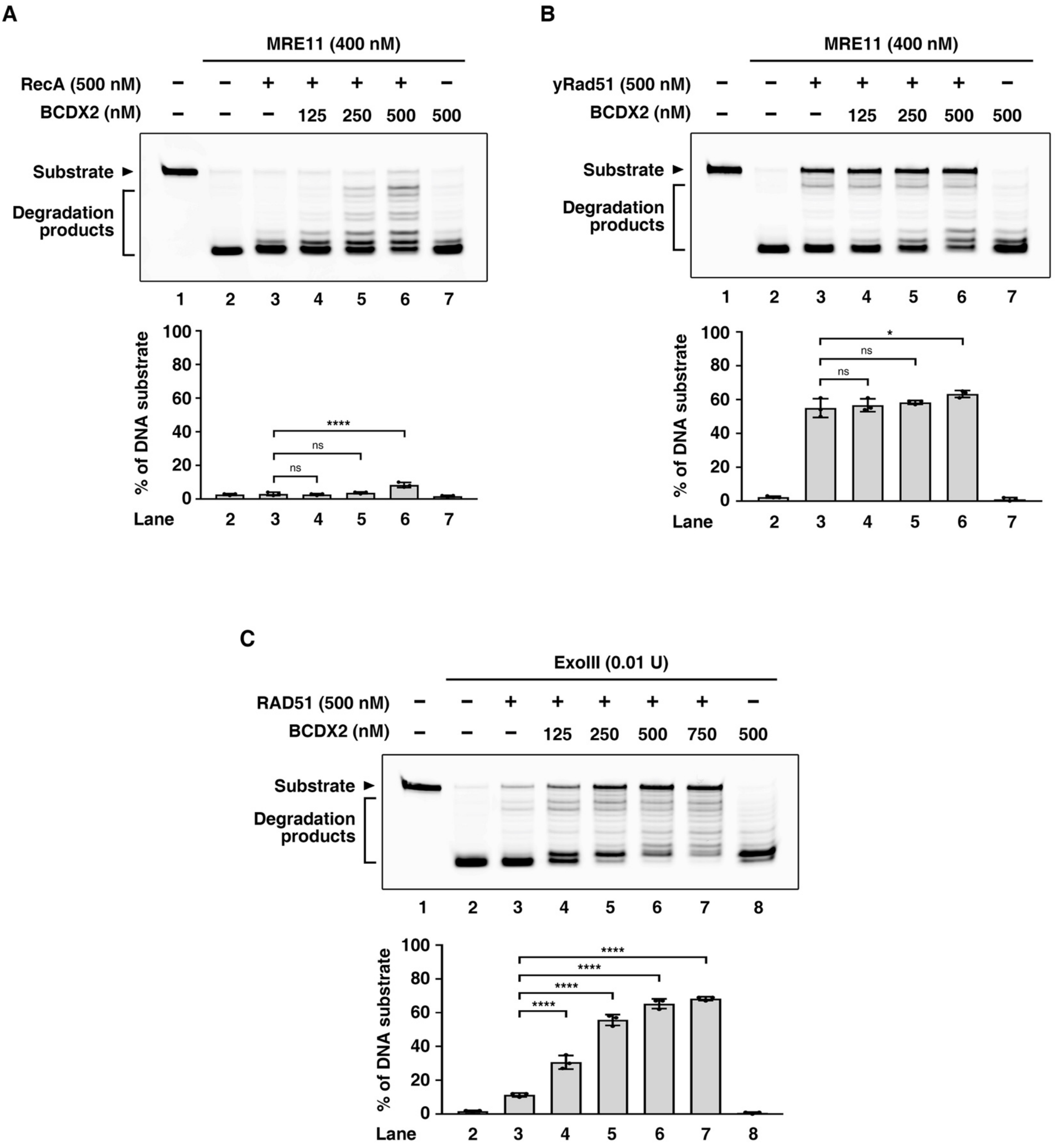

Supplementary Figure S8. The enhanced protection activity by BCDX2 is specific to human RAD51 and non-specifically against various nucleases. (A and B) The MRE11 protection assay was conducted as described above with the indicated amount of RecA (A) or yeast Rad51 (yRad51) (B) along with BCDX2 and MRE11. **(C)** *E. coli* ExoIII protection assay. The indicated concentration of BCDX2, RAD51, and ExoIII was incubated with 58 nM Cy3-labeled 5' overhang DNA substrates at 37 °C for 40 min.

U: abbreviation for a unit. The top panel is the representative gel image; the bottom panel is the quantitative data calculated from at least three independent repeats showing mean ± SD. Statistical significance was determined using one-way ANOVA with Tukey's post hoc test; ns not significant, $*P < 0.05$, $****P < 0.0001$.

Supplementary Figure S9

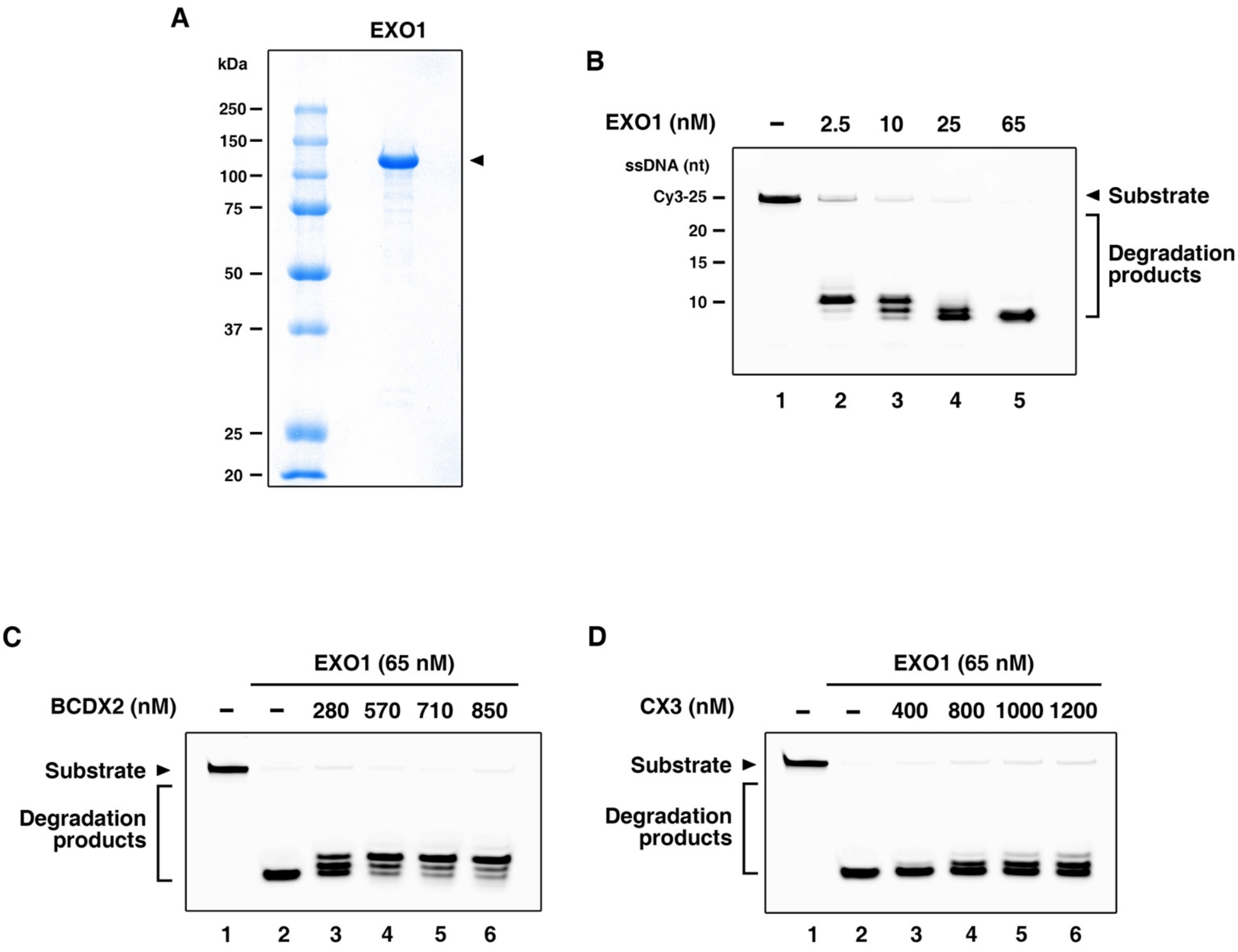

Supplementary Figure S9. BCDX2 and CX3 lack a significant fork protection activity against EXO1 degradation. (A) The purified EXO1 protein (1 μg) was analyzed in a 10% SDS-denaturing polyacrylamide gel with Coomassie Blue staining. **(B)** Analysis of EXO1 nuclease activity. The indicated concentration of EXO1 was incubated with 58 nM Cy3-labeled 3' overhang DNA substrates at 37 °C for 40 min. Reactions were then stopped and resolved in a 27% denatured polyacrylamide gel. **(C and D)** EXO1 protection analysis. The indicated concentration of BCDX2 (C) or CX3 (D) was incubated with EXO1 and the Cy3-labeled 3' overhang DNA substrates (58 nM) at 37 °C for 40 min. Reactions were terminated and resolved as described above.
